# Electrospun nanofibers as versatile interfaces for efficient gene delivery

**DOI:** 10.1186/1754-1611-8-30

**Published:** 2014-12-09

**Authors:** Slgirim Lee, Gyuhyung Jin, Jae-Hyung Jang

**Affiliations:** Department of Chemical and Biomolecular Engineering, Yonsei University, Seoul, 120-749 Korea

**Keywords:** Gene delivery, Electrospun nanofibers, Controlled gene delivery, Tissue engineering, Sustained release

## Abstract

The integration of gene delivery technologies with electrospun nanofibers is a versatile strategy to increase the potential of gene therapy as a key platform technology that can be readily utilized for numerous biomedical applications, including cancer therapy, stem cell therapy, and tissue engineering. As a spatial template for gene delivery, electrospun nanofibers possess highly advantageous characteristics, such as their ease of production, their ECM-analogue nature, the broad range of choices for materials, the feasibility of producing structures with varied physical and chemical properties, and their large surface-to-volume ratios. Thus, electrospun fiber-mediated gene delivery exhibits a great capacity to modulate the spatial and temporal release kinetics of gene vectors and enhance gene delivery efficiency. This review discusses the powerful characteristics of electrospun nanofibers, which can function as spatial interfaces capable of promoting controlled and efficient gene delivery.

**Electronic supplementary material:**

The online version of this article (doi:10.1186/1754-1611-8-30) contains supplementary material, which is available to authorized users.

## Introduction

Gene delivery has emerged as a powerful platform technology for a variety of biomedical applications, including tissue engineering, cancer therapy, and stem cell therapy. Altering genetic information through exogenous stimulation of target cells can accomplish numerous intended goals, such as differentiation of the targets cells into specialized cell types [1–3], activation of apoptosis signals in cancer cells [4, 5], secretion of factors that cause autocrine or paracrine effects in tissues [6–8], or the production of cellular therapeutics [4, 9]. Each of these functions of gene delivery technologies can be essential in a particular case, potentially offering promising strategies for improving the targeted function. Additionally, the identification of new genetic targets or sequences involved in human diseases through the completion of the Human Genome Project has enormously accelerated the progress of gene delivery technologies in numerous applications [10].

Using the full potential of gene delivery in numerous biomedical applications requires a series of toolkits that can help to overcome the limitations associated with gene delivery technologies, the first necessary step prior to employing gene delivery in applications is the selection of suitable gene delivery vehicles, which can be categorized into viral or non-viral vectors, depending on the target applications or cell types. The engineering of versatile gene delivery carriers, which can specifically target clinically valuable cell types (i.e., cancer or stem cells), can avoid immune system effects or toxicities, and can safely pass through complicated intracellular steps to reach the nucleus, is one of the most critical tasks in gene delivery [11–13]. However, the direct administration of gene vectors in liquid formulations to humans, a representative delivery mode, can lead to systemic spread in the body, presumably resulting in risks arising from gene expression in off-target regions [14]. Importantly, direct exposure to viral vectors, which have typically been known to increase gene transfer efficiencies compared with non-viral vectors, might cause severe immune responses against the vectors or even its gene products [15]. Direct injection of gene vectors typically boosts the vector or gene expression dosages in the blood stream or target regions within a short time, possibly leading to cellular toxicities or a short duration of gene expression [7]. Repeated and periodic administration of gene vectors, which can cause pain in patients, may be the only method that can extend the duration of gene expression. These aforementioned concerns about gene delivery are mostly associated with extracellular delivery mechanisms and can raise safety issues, possibly delaying the immediate translation of gene delivery methods into clinical trials. Thus, novel technologies that can modulate gene delivery routes or profiles within extracellular environments must be used to facilitate the successful translation of gene delivery for human clinical use. Combining gene delivery with biomaterial systems has been commonly discussed as a powerful strategy that can provide opportunities to more effectively apply gene delivery for many biomedical applications [6, 7, 13, 16].

In this review, we primarily focus on the strategy that combines gene delivery with electrospun nanofibers as one strategy among all of the interdisciplinary approaches of gene delivery with biomaterials, which have been typically employed as tissue engineering scaffolds [17–19], microparticles [20, 21] and nano or micro devices [22, 23]. This strategy addresses the concerns about both gene delivery and potential applications, and these nanofibers can be easily fabricated using a cost-effective method. Electrospun nanofibers have been extensively explored as spatial templates that can effectively mimic the structure or functions of extracellular matrices (ECMs), thereby working as highly effective interfaces that can retain cellular morphologies and efficiently deliver biomolecules to target cell types [24, 25]. Thus, electrospun nanofibers have great potential as a physicochemical guide that can be used for numerous biomedical applications, including tissue engineering and drug or gene delivery [24, 26]. This review will primarily discuss the powerful characteristics of electrospun nanofibers as spatial templates for gene delivery. Finally, successful employment of the combinatorial approaches of gene delivery with electrospun nanofibers will be classified depending upon the application, including tissue engineering, cancer therapy, and stem cell studies.

## Electrospun nanofibers as versatile spatial templates

**Figure 1:**
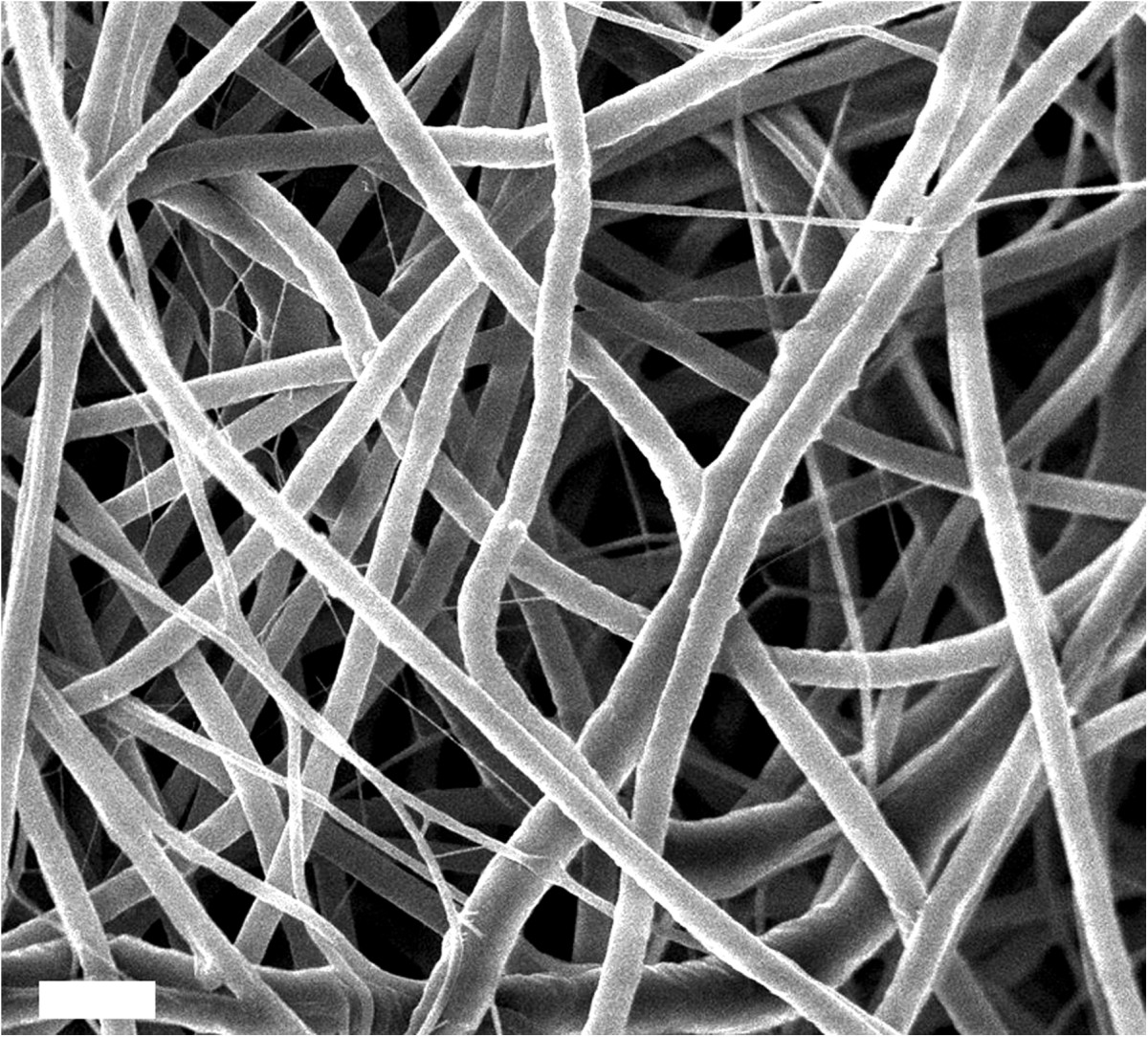
**ECM-analogue morphology of electrospun PCL nanofibers imaged using scanning electron microscopy (SEM).** The scale bar indicates 1 μm. The image was reproduced from [48], Copyright 2011, with permission from Elsevier.

Electrospinning is a versatile method for fabricating ultrafine polymeric nanofibrous structures through electrostatic interactions (Figure 1). Deposition of the resultant fibers on grounded collectors can produce non-woven fibrous matrices with high surface-to-volume ratios and diameters ranging from nanometers to micrometers [27–29]. One of the highly advantageous aspects of using electrospun matrices as building blocks for numerous biomedical applications is the feasibility of manipulating the physical and chemical characteristics of the resultant fibrous structures. Specifically, the surface morphology [30, 31], mechanical strength [32, 33], fiber orientation [34–36], and inner structure of the fibers [37–39] can be diversified by simply adjusting the various fabrication parameters, such as collector designs and nozzle alignments. Additionally, a variety of materials, including metals [40, 41], ceramics [42–44], synthetic polymers [33, 45–47], peptides [48, 49], and viral solutions [50], can be applied to produce electrospun nanofibrous structures. Conventional electrospinning techniques typically generate two-dimensional sheet-like shapes. In contrast, advanced electrospinning tools, such as layer-by-layer deposition [45, 46], E-beam etching [51, 52], selective leaching [33], and plasma treatment [53, 54], have recently been developed to produce well-defined or sophisticated three-dimensional fibrous structures, ultimately further expanding the scope for numerous biomedical applications.

## Electrospun nanofibers for gene delivery

Owing to these multiple merits, highly porous fibrous structures have recently been employed as powerful templates for delivering genes of interest for various purposes (e.g., tissue engineering, cancer treatment or stem cell studies) in a spatially or temporally regulated manner [24–26]. Non-viral gene vectors, such as naked plasmid DNA or DNA/polyplexes, have primarily been incorporated within or onto electrospun nanofibers, presumably because of their ease of production or their capacity to maintain the intact properties compared with viral vectors [55–57]. The use of viral vectors, which are created by stripping the viral genomic sequences and subsequently packaging the genes of interest into the viral capsid, has recently been integrated with electrospun nanofibers, primarily to further increase the gene delivery efficiency or extend the duration of gene expression [30, 33, 48].

To ensure that electrospun fibers can be used as gene delivery templates, the lack of harmful effects of two major processing features on the bioactivities of gene vectors must be confirmed; the processing typically includes direct contact between gene vectors and organic solvents and exposure to a high electric voltage. No systematic studies have been performed to thoroughly investigate the detrimental effects of the electrospinning processes on the activities of gene vectors. However, no substantial loss of the capabilities of gene vectors as a result of the relatively harsh electrospinning processes has been observed in many previous studies [47, 48, 58]. The methods for loading gene vectors in electrospun nanofibers can be classified into two categories, which are summarized in Table 1: encapsulating gene vectors within the fiber interiors during the fiber production process, and immobilizing gene vectors onto the fiber exterior after the process. The former methods, which incorporate gene vectors within structures by simple mixing with the polymer solution, emulsification, and co-axial electrospinning, primarily focus on the controlled release of gene vectors by modulating the physical or chemical properties of fibrous constructs. In the latter methods, gene vectors are typically associated with electrospun nanofibers after the completion of all the fabrication procedures, and the main goal is to prevent the possible harmful effects of organic solvents or a high electric field on the activities of gene vectors and thus further increase gene delivery efficiencies. Additionally, post-adsorption approaches can be adjusted to allow the spatial control of gene delivery by using additional technological tools, such as surface chemistry [33, 46, 59] and vector modifications [60].

**Table 1:** **Classification of electrospun nanofiber-mediated gene delivery approaches**

Vector loading methods	Vectors	Genes	Target cells	Results	Ref
Vector encapsulation	Virus (Ad)	GFP, RFP	HEK293T	• Sustained and controlled viral release for 30 days.	[30]
				• Localized gene expression from electrospun scaffolds.	
	Plasmid DNA	β-Gal, GFP	MC3T3-E1	• Gene expression by the released DNA 48 h after seeding	[47]
				• Burst release of majority of encapsulated plasmid DNA within 30 minutes.	
		Cdk2i, EGFPi	MCF-7 cell	• Sustained release over 21 days	[115]
				• ~40% decrease in proliferation of breast cancer cells compared with control scaffold	
		EGFP	Rat fibroblasts	• Extended release of pDNA and transgene expression over 60 days.	[55]
	Virus (AAV)	GFP	NIH3T3	• Sustained viral release for 7 days.	[48]
				• Maintained transgene expression (>90%) on the scaffolds for 7 days.	
	Plasmid DNA/chitosan nanoparticle	BMP-2	hMSC	• Sustained release for 45 ~ 55 days	[56]
				• DNA/chitosan nanoparticles encapsulated electrospun scaffolds as a favorable DNA delivery device with regard to cell transfection efficiency and cell viability	
	Plasmid DNA/LEL polyplex	β-Gal, GFP	MC3T3	• Transgene expression on DNA-incorporating electrospun scaffolds 24 h after seeding.	[57, 80]
				• Sustained release for 7 days.	
	siRNA/CPP polyplex	Col1A1 silencing	Human dermal fibroblasts, *in vivo*	• Prolonged *in vitro* gene silencing duration by 2 ~ 3-fold.	[58]
				• *In vivo* gene silencing for 4 weeks.	
	Plasmid DNA/PEI	EGFP	NIH3T3	• Controlled release time from 6 days to 25 days by internal structures and porogens.	[64]
				• 10-fold increased gene expression on the scaffolds compared to simple pDNA/PELA blends.	
		VEGF/eGFP & bFGF/eGFP	HUVEC	• Sustained release for 4 weeks	[65]
				• Significantly higher vessel densities	
		bFGF/GFP	BEF, *in vivo*	• Sustained release for 26 days	[84]
				• 4 ~ 6-fold increased bFGF expression compared with post-electrospinning delivery after 7 day incubation	
				• Significantly higher wound recovery rate compared with post-electrospinning delivery	
	siRNA/chitosan polyplex	EGFP silencing	EGFP expressing human lung carcinoma cell lines	• Sustained and controlled delivery for 30 days.	[67]
				• Prolonged *in vitro* gene silencing duration by 3 ~ 4-fold compared to the bolus delivery.	
	siRNA	GAPDH silencing	HEK293, NIH3T3	• Sustained release of siRNA for 28 days.	[68]
				• Gene silencing on scaffolds in presence of additional transfection agents.	
				• Enhanced gene silencing capability with additional transfection agents in the media.	
	siRNA/transfect-ion reagent complex	GAPDH silencing	NIH3T3	• Sustained release of siRNA and gene silencing on the scaffolds for at least 28 days.	[69]
				• Improved gene silencing capability with transfection agents supplemented in the media.	
	Solid-in-oil dispersion of plasmid DNA	Luciferase	N/A	• Release profile controlled by degrading rates of fibers.	[81]
				• 10-fold increases in functional integrity of released pDNA compared to mixed mesh.	
	Plasmid DNA/calcium phosphate nanoparticle	VEGF/eGFP & bFGF/eGFP	HUVEC, hAoSMC	• Sustained release for 4 weeks	[83]
				• Significantly higher densities of blood vessels and mature vessels	
Vector immobilization	Virus (AAV)	GFP, Luciferase	HEK293T	• Three-dimensional and uniaxially aligned transgene expression	[33]
				• 4-fold enhanced transgene expression levels compared to 2D electrospun scaffolds.	
	Plasmid DNA	EGFP	Glioblastoma cells	• Transgene expression by the released DNA from the fibers (maximum transfection efficiency > 90%).	[45]
		Luciferase	COS-7	• Retained gene expression on the fibers for 5 days after seeding.	[46]
				• 2-fold increased gene delivery efficiency of electrospun fibers over that of flat films.	
		GFP, Dsred	HEK293, MSC, *in vivo*	• 10-fold increase in gene expression intensity compared to PCL fibers *in vivo*.	[59]
		EGFP-N1	NIH3T3, i*n vivo*	• MMP-2 responsive release of DNA	[66]
				• Significantly enhanced gene expression in wound tissue compared to naked DNA delivery	
		Luciferase, KGF	NIH3T3, *in vivo*	• Sustained expression for 7 days	[76]
				• 65% smaller epithelial gap in KGF scaffold treated wounds than in untreated wounds	
		hEGF	HDF, *in vivo*	• MMP-2 responsive release of DNA	[85]
				• Approximately 2-fold increased wound closure compared with non-treated wounds	
		EGFP	MC3T3-E1	• Controlled gradients of pDNA concentration and gene expression level by spatially regulating rates of chemical reactions.	[98]
	Virus (AAV)	GFP	HEK293T	• Patterned and localized gene vectors and gene expression on the scaffolds.	[60]
				• 2-fold increase in transfection efficiency compared with unmodified virus delivery.	
	Plasmid DNA/liposome	RUNX2/eGFP	hBMSC	• Long-term gene expression for 21 days	[77]
				• Improved osteogenic differentiation of stem cells	
	siRNA/PEI polyplexes & siRNA/ transfection reagent complex	TSP-2 silencing	hAoSMC	• Down-regulated TSP-2 mRNA expression	[78]
	Plasmid DNA/chitosan nanoparticle	BMP-2	*in vivo*	• Different bone healing performance depending on the loading methods	[82]
				• Improved bone healing for DNA/chitosan nanoparticles adsorbed electrospun scaffolds at 4 weeks of treatment	
	siRNA	MMP-2 silencing	HDF, *in vivo*	• MMP-2 responsive release of DNA	[86]
				• Faster wound recovery rate compared with siRNA solution delivery	
	Plasmid DNA/ssPEI	Luciferase, RFP, VEGF	H9C2 myoblastic cell	• Enhanced transfection efficiency compared to bolus delivery	[87]
				• Successful expression of the VEGF gene in the cells	
	siRNA/ transfection reagent complex	REST silencing	NPC	• Enhanced neural marker expression and neuronal differentiation	[88]

### Advantageous aspects of electrospun nanofibers for gene delivery

Combining gene delivery with engineered polymeric biomaterials has been regarded as an indispensable strategy to increase delivery efficiencies and modulate gene delivery kinetics in a spatial and temporal manner. Genes delivered via a classic method (i.e., direct administration of a liquid formulation) diffuse freely within the body and thus suffer from the regulation of gene delivery rates or the localization of gene expression within a designated region [13, 14]. In contrast, manipulating the physical or chemical properties of polymer matrices or modulating the molecular interactions of gene vectors with polymer materials can readily tune the release profiles of gene vectors, which can range from a few hours to more than months [6, 8]. The sustained release of gene vectors from polymeric templates can increase their residence time within the cellular microenvironment, potentially enhancing the gene transfer efficiency and extending the duration of gene expression [16]. Importantly, the extremely large surface-to-volume ratios and the ECM-analogue nature, which are unique properties of electrospun nanofibrous structures, make nanofibers powerful alternatives for maximizing the capabilities and efficiency of gene delivery in a variety of biomedical fields [25, 61].

The high porosity of electrospun nanofibers can facilitate increased cellular contacts with well-distributed gene vectors within or over large surface areas, thereby allowing more opportunities to internalize gene vectors across the cellular membrane. Furthermore, the pores of electrospun nanofibers are typically produced with interconnected open structures, which can improve the cellular penetration into fibrous interiors and increase the gene vector loading capacities, thereby increasing in the gene delivery efficiencies [62]. For example, the adsorption of adeno-associated viral (AAV) vectors on the surface of three-dimensional fluffy fibrous structures, whose specific pore volume was 4-times greater than that of two-dimensional electrospun mats, allowed a significant improvement in cellular transduction efficiencies compared with vectors associated with flat non-porous polymeric systems [33]. Consistently, immobilizing plasmid DNA on highly porous poly(lactic) acid (PLA) fibrous matrices resulted in approximately 1.5-fold increased gene delivery efficiencies compared with those for non-porous PLA films [46], confirming the superior contribution of nanofibrous pore structures to gene delivery.

### Strategies for controlling electrospun nanofiber-mediated gene delivery

Owing to the ease of tuning structural variations of electrospun nanofibers, the capability of these fibers to mediate controlled and sustained gene delivery has been recognized as the most representative feature of electrospun nanofibers compared with other existing polymeric gene delivery templates. It is well known that variations in polymer degradation through hydrolysis and alterations of diffusion routes through porous structures have been key design parameters that can vary the release kinetics of biomolecules encapsulated within or adsorbed on polymeric templates [63]. In addition to these conventional ways to control release profiles, additional design variations in electrospun nanofibers have been explored to diversify the kinetics of the release of gene vectors from the fibrous structures. For example, the structural characteristics of individual fibers can be altered by changing the formulation of the core-sheath structures or by modifying the surface properties as an alternative strategy to vary the release rates of incorporated or adsorbed gene vectors from the electrospun fibers or to further increase delivery efficiencies [64, 65]. The following section describes versatile methods capable of mediating controlled gene delivery from electrospun nanofibers; these methods were classified by the vector-loading approaches, in which the vector is encapsulated into the interior (Controlled release from nanofibers by encapsulating gene vectors and Core-sheath formulations for controlled release) or immobilized onto the exterior of nanofibers (Substrate-mediated gene delivery using electrospun nanofibers).

#### Controlled release from nanofibers by encapsulating gene vectors

**Figure 2:**
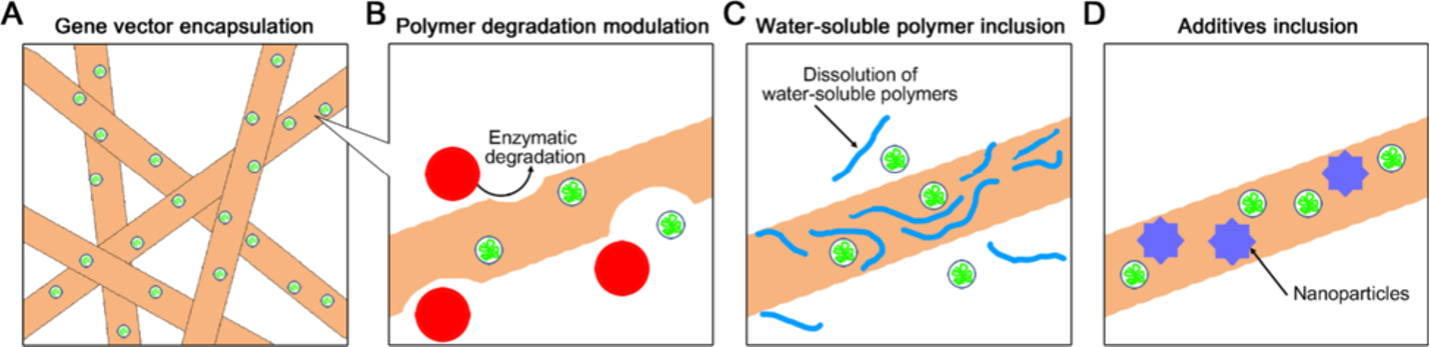
**Blending approaches for controlled gene delivery. (A)** Schematic illustration demonstrating gene vector encapsulations within electrospun fibers. The right three panels display strategies for controlled delivery using the blending-approaches via **(B)** modulating the environmental conditions, **(C)** including water-soluble polymers and **(D)** providing additives **(D)**.

The encapsulation of gene vectors within electrospun nanofibers for subsequent diffusion through porous routes can result in the sustained release of gene vectors, as well as controlled delivery via manipulations of the physical or chemical properties of the fibrous structures. Gene vectors can be incorporated within the inner space of fibrous structures, as illustrated in Figure 2, by simply mixing aqueous DNA buffers with polymer solutions in organic solvents, followed by electrospinning the nanofibers. The first trial involving loading gene vectors into electrospun nanofibers was performed by blending Tris-EDTA buffer solution containing plasmid DNA, which encoded β-galactosidase driven by the cytomegalovirus (CMV) promoter, with the block-copolymers of PLA and poly(ethylene glycol) (PEG) dissolved in N,N-dimethyl formamide [47]. In this study, the activity of the plasmid DNA was stably maintained during the encapsulation and electrospinning process. To reduce the steric repulsion between the hydrophobic PLA solution and plasmid DNA in the polar aqueous buffer, the hydrophilic polymer PEG was included in the mixture prior to electrospinning. The resultant DNA-blending approach within the fiber interior spaces containing the hydrophilic PEG resulted in a rapid DNA release within 2 hours, followed by a high level of gene expression at 48 hours post-transfection. The sustained release of the plasmid DNA, whose release kinetics was determined by both the pore morphologies and the contents of the copolymers used to produce the nanofibers, was maintained for at least 20 days, demonstrating the efficacy of DNA-blending approaches for efficient gene delivery as well as for an extended duration of gene delivery.

The release profiles of gene vectors within the inner space of nanofibers were readily regulated using environmental factors that can degrade polymer matrices (Figure 2B). It is well known that polymer degradation patterns, which can be categorized into either surface erosion or bulk degradation, can determine whether the release kinetics of incorporated gene vectors follow a sustained mode or burst release mode [63]. The addition of proteinase K into the release buffer accelerated the enzymatic degradation of polyester-based biodegradable polymers, such as poly(L-lactide-co-ϵ-caprolactone) (PLCL) or poly(caprolactone) (PCL), resulting in a rapid burst within a short time period [66]. Approximately the total quantity of the plasmid DNA incorporated in the PCL matrices could be released within 10 hours when the releasing buffer contained proteinase K, whereas quite slow release rates of plasmid DNA were observed without the enzyme in the buffer solution. Similarly, varying the acidity of the releasing buffers fixed the acidic or alkaline hydrolysis patterns of poly(D,L-lactide-co-glycolide) (PLGA), which typically results in the bulk or surface erosion of PLGA, respectively [67]. Consequently, the alteration of the polymer erosion modes via manipulations of the environmental acidity acted as a crucial parameter to regulate the release mechanisms of small interfering RNA (siRNA) encapsulated within PGLA nanofibers. The resultant sustained delivery of siRNA from the PLGA fibrous matrices, which were slowly degraded in acidic environments, prolonged the gene silencing effects for up to 30 days.

Another key factor that can modulate the release modes of gene vectors encapsulated within nanofibers is the inclusion of additives within fibers (Figure 2C and 2D). Water-soluble additives that are included within the hydrophobic polymer fibers can function as porogens that allow the incorporated gene vectors to readily pass through the routes, whose dimensions are newly expanded via the dissolution of the additives in aqueous solution (Figure 2C). For example, the incorporation of hydrophilic PEG content within hydrophobic fibers provided favorable hydrophilic traces that increased the affinity for gene vectors or siRNA complexes, possibly facilitating the diffusion of the incorporated vectors from the fibrous matrices [68]. Additionally, the addition of hydroxylapatite (HAp) nanoparticles to PLGA nanofibers regulated the DNA release rates and promoted cellular adherence on the fibers as well (Figure 2D) [56]. The incorporation of HAp nanoparticles triggered the association of DNA/chitosan complexes with PLGA nanofibers during the fiber fabrication process, substantially improving the DNA loading efficiencies. The inclusion of the hydrophilic inorganic nanoparticles, which altered the mechanical properties of the composite fibers, including their strain–stress behaviors and glass transition temperatures, accelerated the release of DNA/chitosan complexes and enhanced the gene delivery efficiencies as well as the cellular viabilities.

Altering the compositions of polymeric materials comprising nanofibers can be an additional option to tailor the release profiles of gene vectors, as well as the delivery efficiencies. The copolymerization of ethyl ethylene phosphate (EEP) with caprolactone facilitated the incorporation of gene vector elements into the inner space of nanofibers and resulted in a sustained release of siRNA complexes that exceeded 40 days [69]. The co-encapsulation of small-interfering RNA with either a transfection reagent or cell-penetrating peptides within poly(caprolactone-co-ethylethylene phosphate) (PCLEEP) nanofibers resulted in increased gene silencing efficiencies and extended the duration of gene silencing to over 14 days, thus prolonging the expression by approximately 2-3-fold compared with that for bolus delivery [58]. Consequently, the sustained delivery of siRNA suppressing the production of collagen type I substantially reduced the fibrous capsule thickness adjacent to nanofibrous scaffolds that were implanted subcutaneously. Additionally, blending the PCL solution with elastin-like polypeptides (ELP) in hexafluoro-2-propanol (HFP) triggered the release of AAV vectors and prolonged the viral delivery to more than 14 days [48]. In this study, the phase transition properties of ELPs at different temperature enabled versatile AAV release profiles at various temperatures. Taken together, these findings show that the presence of hydrophilic portions within hydrophobic building blocks can facilitate the porogen-assisted release of incorporated agents. Furthermore, in addition to these factors that affect the release profiles, the inclusion of hydrophilic materials into fibrous matrices can offer additional promising features, such as improved biocompatibility and cellular attachment.

#### Core-sheath formulations for controlled release

**Figure 3:**
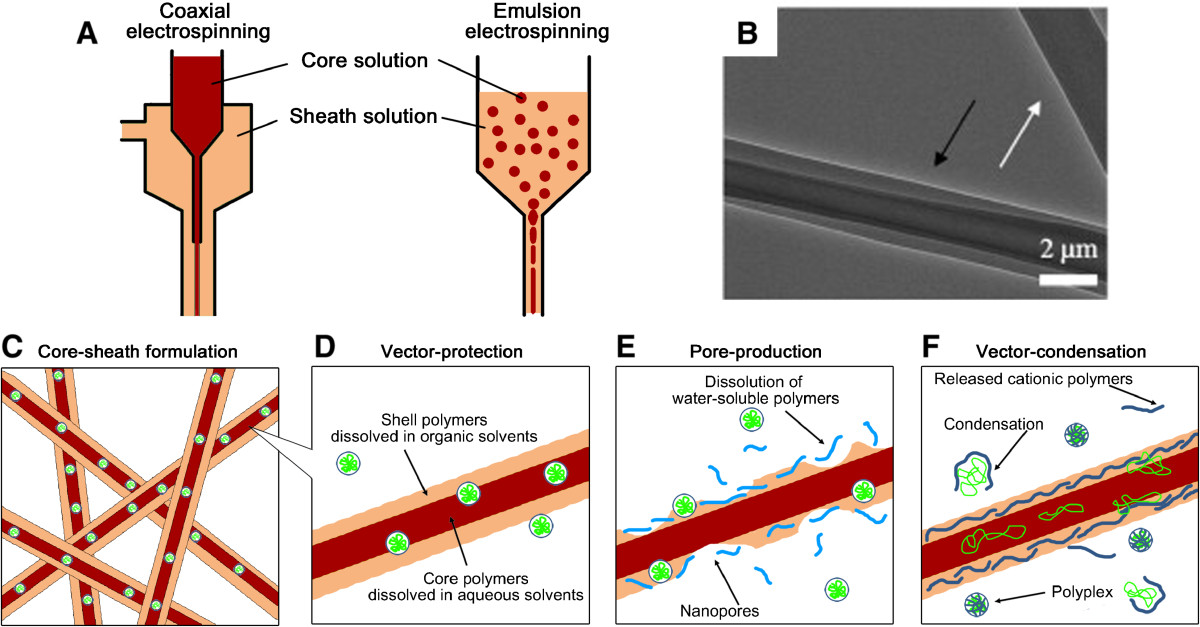
**Core-sheath electrospun nanofibrous systems for controlled gene delivery. (A)** Two representative methods to form core-sheath electrospun nanofibers: coaxial (left) and emulsion (right) electrospinning. **(B)** Transmission electron microscopy (TEM) image of an individual core-sheath nanofiber fabricated using coaxial electrospinning. Core and sheath are composed of viruses dispersed in Minimal Essential Medium and PCL, respectively. Scale bar is 2 μm. Reprinted from [30], Copyright 2009, with permission from Elsevier. **(C)** A scheme depicting gene vector encapsulation within the core layer for controlled release. The core-sheath fibrous formulations contribute **(D)** to preventing the direct contact of gene vectors in the core layer with organic solvents in the sheath layer, **(E)** to regulating delivery modes by producing porous sheath layers, and **(F)** to enhancing delivery efficiencies by modifying the sheath layers with polycationic polymers.

A notable structural feature of the electrospinning process is its capability to produce a core-sheath structure within individual fibers, where multiple biomolecules at each layer can be designed to diffuse out sequentially (Figure 3). The electrospun nanofibers with the core-sheath structures can be fabricated using co-axial electrospinning (Figure 3A) [33, 39] or the emulsion electrospinning technique (Figure 3B) [64]. The core-sheath structures, whose representative morphology is demonstrated in Figure 3C, have been typically produced for the following: i) the protection of gene vectors from direct exposure to organic solvents and ii) the controlled release of gene vectors residing in core layers through modifying the shell structures. The inclusion of gene vectors in hydrophilic core-layers followed by encapsulation with hydrophobic shell-layers in organic solvents can prevent the direct contact of gene vectors with organic solvents (Figure 3D). Differences in the diffusion pathways of gene vectors through two layers composed of different materials can alter the release rates of the incorporated gene vectors in each layer, which have already been observed in many drug delivery studies using core-sheath structures [70, 71]. Unfortunately, the sequentially controlled release of multiple gene vectors from each core-sheath layer has not been explored yet. Taken together, these possibilities for the integration of gene delivery technologies into the core-sheath fibrous matrices can provide an efficient means to control the sequential release of multiple vectors and can simultaneously protect gene vectors in the core-layer against the relatively harsh processes.

The presence of porogens in the shell layers can facilitate the sustained and controlled release of gene vectors incorporated in the core layers and can also extend the duration of gene delivery (Figure 3E). The production of shell layers with higher levels of PEG, which was deposited in the shell layer by varying the molecular weights and concentrations of the PEG, led to the rapid release of the plasmid DNA that was complexed with poly(ethylenimine) (PEI) [64]. The dissolution of PEG in aqueous environments (i.e., cell culture medium) might provide more space for the diffusional passage of gene vectors in the core layers to the outer spaces. He et al. used dual gene vectors that encoded different inducible factors (i.e., vascular endothelial growth factor (VEGF) and basic fibroblast growth factor (bFGF)) and loaded them together into the core poly(D,L-lactide)-poly(ethylene glycol) (PELA) layers that were encapsulated by a sheath layer containing PEG [65]. The sustained release of these multiple vectors from the core layer through the PEG-assisted routes in the sheath layer synergistically promoted mature blood vessel formation. In this particular case, the pores exist separately in the exterior layer, and the burst of gene vectors at the initial time points may be avoided, which is not possible in cases in which the pores coexist with vectors in the same layer (i.e., single fibers). This difference can be demonstrated by comparisons of the parallel data from several studies [56, 65]. Importantly, the enlarged surface areas in the shell layers might create room for cell migration toward the inner spaces, where cells can encounter the gene vectors released from the core layer.

The variations in the PEG contents in the shell layers demonstrated the distinctive controlled release profiles of viral vectors that were encapsulated within the core layers. The release rates of adenoviral (Ad) vectors encapsulated within co-axially produced PCL fibers were dependent on the PEG concentrations in the shell layers [30]. As the PEG contents increased in the shell layers, highly rapid viral elution from the fibers in a short time period (~ a week) was observed, but the inclusion of intermediate quantities of PEG resulted in a gradual release with almost constant rates over 30 days. As a result, the continuous supply of Ad vectors to HEK293T cells that were cultured on highly porous PCL core-sheath fibers persistently induced high levels of cellular transduction. However, non-porous Ad-encapsulated PCL fibers exhibited almost no viral release over a month, resulting in extremely low levels of transduction throughout the time points. Ad capsid PEGylation, which was primarily performed to protect Ad vectors from immune rejection, has been shown to inherently reduce transduction efficiencies [72]. However, the extended duration of the substantially increased gene expression by PEG-assisted Ad delivery from PCL fibers demonstrates the superior capabilities of polymeric gene delivery compared with direct delivery approaches.

The core-sheath fibrous structures can provide multiple physical spaces for separately delivering dual factors, which possess individual roles (Figure 3F). Co-axially electrospun nanofibrous matrices were produced by incorporating plasmid DNA in the core PEG layer, along with a derivative of PEI conjugated with hyaluronic acids (HA-PEI) in the shell layer that contained PCL polymers dissolved in a chloroform and methanol mixture [55]. The inclusion of plasmid DNA in the core PEG region was likely attempted to minimize the exposure of the plasmid to organic solvents in the shell layer and to prevent additional processes (i.e., lyophilization) that could ultimately reduce the activity of the plasmid DNA [73, 74]. The lyophilization process was typically required in blending approaches to uniformly spread DNA powders in hydrophobic polymer solutions prior to electrospinning. Interestingly, the cationic polymer HA-PEI in the outer sheath layer self-assembled with the negative plasmid DNA vectors that were released from the core layer, thereby enhancing the efficiencies of cellular internalization in a rat fibroblast cell line [55]. The coordination of the release profiles of both factors (i.e., plasmid DNA and HA-PEI), whose release rates were further tuned by additional parameters (e.g., concentrations or molecular weights of the polymers in each layer), resulted in persistent gene expression over 60 days. The increases in the delivery efficiencies were significantly greater than those for PCL matrices containing pDNA alone. Owing to the capacity to modulate the release profiles of multiple biomolecules in each layer, electrospun nanofibers with core-sheath structures have great potential as a platform template for tailoring the delivery kinetics of multiple gene vectors, potentially leading to the broad application of these fibers in numerous biomedical fields.

#### Substrate-mediated gene delivery using electrospun nanofibers

An alternative approach capable of delivering gene vectors from electrospun nanofibers is to immobilize gene vectors onto the surfaces of completed nanofibrous structures; this technique is termed substrate-mediated delivery. This approach can block any attempts to expose gene vectors to both organic solvents and a high electric field because the gene vectors are adsorbed on the surface of fibrous constructs in the last step after the manipulation of the fibrous constructs is completely finished. Thus, the relatively harsh processing steps could have no harmful effects on the bioactivities of gene vectors. Importantly, this delivery method can place gene vectors in close proximity to the desired location within the cellular microenvironment and can extend the residence time of the gene vectors within the boundary layers; thus, this method can overcome mass transfer limitations to deliver the genes of interest to target cells [7, 75]. Increasing the extent of physical contact of gene vectors with target cells that are seeded on fibrous structures is thought to significantly increase the delivery efficiencies of gene vectors, which is a primary goal of the substrate-mediated delivery systems. Gene vectors can be immobilized on the fibrous interfaces primarily by either simple random adsorption [45, 46, 59, 76] or specific adherence [77, 78]. Importantly, tuning the interactions of gene vectors with fibrous surfaces by altering the surface chemistry or by modifying the vectors themselves can result in the spatially and temporally controlled delivery of gene vectors, which can be a unique feature of substrate-mediated gene delivery. This section classifies substrate-mediated gene delivery using electrospun nanofibers according to the method for immobilizing the gene vectors on the fiber surfaces.

##### Non-specific adsorption of gene vectors on the fibrous surfaces

**Figure 4:**
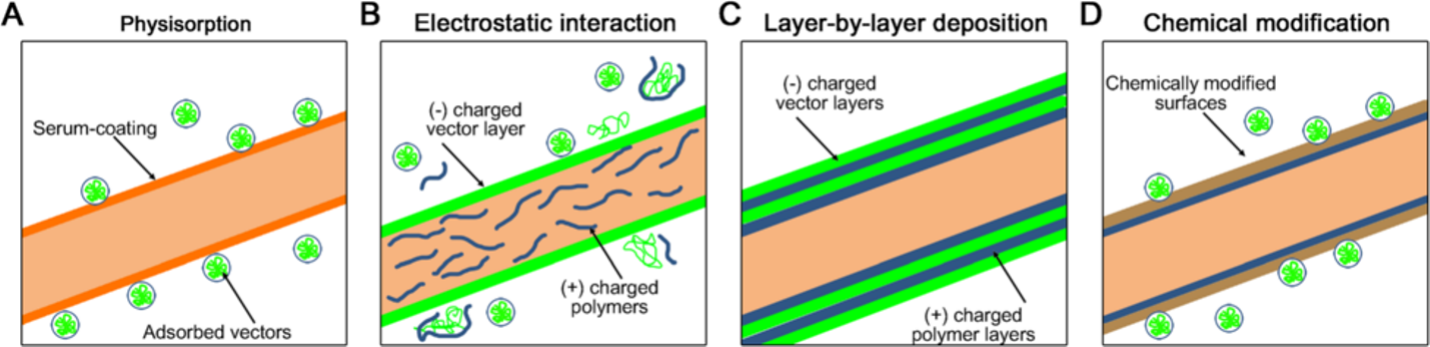
**Substrate-mediated gene delivery from electrospun nanofibers.** Gene vectors can be simply adsorbed on the fibrous surfaces via **(A)** physisorption, **(B)** electrostatic interaction, and **(C)** layer-by-layer deposition techniques. **(D)** Gene vectors can be specifically immobilized on the chemically modified fibrous surfaces to further enhance the mutual interactions between vectors and fibers.

The most representative method of immobilizing gene vectors on electrospun fibers is non-specific random adsorption, which is typically accomplished using van der Waals, hydrophobic, and electrostatic interactions between gene vectors and the fiber surfaces (Figure 4A) [7, 13, 79]. Zhang et al. adsorbed plasmid DNA on PCL nanofibrous matrices blended with a cationic PEI through the electric attraction between the negatively charged DNA and the cationic PEI (Figure 4B) [59]. While the non-specific adsorption onto the PCL fibers without the inclusion of PEI resulted in substantially lower quantities of plasmid DNA, charging the PCL fibers with cations by blending with PEI substantially increased the DNA adsorption and yielded high transfection efficiencies in both human embryonic kidney cells and mesenchymal stem cells. Similar to the substrate-mediated delivery of non-viral vectors, the non-specific random adsorption of AAV capsid particles on the serum-coated three-dimensional moldable PCL fluffy matrices resulted in highly potent gene expression throughout the entire volumetric matrices; this process led to approximately 5-fold increased luciferase expression compared with that on two-dimensional PCL fibrous sheets [33]. This observation confirms the crucial contribution of large-surface, porous structures to higher gene delivery efficiencies.

Producing multiple DNA layers on fibrous surfaces was proposed as a means to increase gene delivery (Figure 4C) [45, 46, 76]. Layer-by-layer (LBL) DNA films were constructed by iterative accumulations of plasmid DNA on fibrous matrices, which were designed to possess cationic properties by electrospinning polycationic poly(β-amino ester) (PBAE) poly(1,4-butanediol diacrylate-co-4-amino-1-butanol) end-capped with 1-(3-aminopropyl)-4methylpiperazine (447) [45]. The concentration of the polymer 447 was one of the key parameters that could influence the total quantity of the multiple DNA layers and ultimately worked as a crucial factor to tune the DNA release profiles and improve the gene delivery to primary human glioblastoma cells. Interestingly, the number of DNA layers that accumulated on the fibrous surfaces was directly related to the level of transgene expression, whose levels improved as the number of DNA layers increased [76]. Kobsa et al. formed multi-layered DNA/PEI films on electrospun fibrous matrices composed of PLA or PCL; this process resulted in persistent luciferase expression for at least 7 days and consequently accelerated the wound re-epithelialization, keratinocyte proliferation, and granulation response [76]. These combinatorial approaches with the LBL technique will expand the scope of substrate-mediated gene delivery systems for applications that typically require long-term gene expression.

Electrospun nanofiber-mediated gene delivery approaches using naked plasmid DNA typically require additional process, such as condensing the plasmid DNA to further increase the delivery efficiencies; the delivery capability for naked plasmid DNA is substantially lower than that for viral vectors. Different methods were employed to decrease the size of plasmid DNA upon electrospinning: i) solvent-induced condensation [57, 80], ii) emulsion-induced condensation [81], and iii) polycation-induced condensation [56, 82, 83]. Exposing DNA to a co-solvent system composed of 94% DMF and 6% TE buffer resulted in a transition from a random coil structure to a structure containing both coil and globule features [57, 80], and the resultant DNA complexes that were released from the PLGA fibrous matrices showed significantly increased cellular transfection compared with that for non-complexed DNA. Additionally, a solid-in-oil (S/O) emulsion-induced condensation, which formed a solid phase with lyophilized DNA in cyclohexane, maintained the activity of DNA upon electrospinning with dichloromethane containing PLCL [81]. For the resultant S/O nanodispersion, the bioactivity of gene vectors improved approximately 12-fold improved compared with that for the system produced by simple mixing; this improvement potentially provides potent gene vectors that can increase delivery efficiencies [81]. Polycation-induced condensation, which is performed prior to electrospinning, is the most widely used method for condensing non-viral vectors to further increase transfection efficiencies [56, 82, 83]. The complexation of plasmid DNA with PEI followed by electrospun fiber-mediated delivery was superior to bolus delivery of DNA complexes at increasing transfection efficiencies for extended time periods [84]. In addition to acting as a DNA-condensing agent, PEI has been combined with electrospun fibers as surface-coating [46, 66, 85, 86] or blending agent [59, 64, 65, 76, 78, 84, 87]; all of these functions were utilized to develop the most efficient fibrous systems to release non-viral vectors by balancing the release profiles of DNA complexes, the cytotoxicity of PEI itself, and the interaction with cellular membranes [11, 59].

##### Surface immobilization of gene vectors for substrate-mediated gene delivery

The chemical modification of fibrous surfaces is an efficient way to adhere gene vectors onto fibrous interfaces, through increasing the specificity of the fibers for the gene vectors (Figure 4D) [77, 78, 88]. Monteiro et al. used the affinity of liposomes with thiol groups exposed on PCL nanofibrous matrices to specifically attach DNA/liposome complexes onto SH-functionalized surfaces [77]. The specific immobilization of the DNA/liposome complexes resulted in the prolonged expression of runt-related transcription factor 2 (RUNX2) from human bone-marrow-derived mesenchymal stem cells (hBMSCs) and increased the viability of the hBMSCs on the modified PCL fibers. In another study, PCL fibers that were functionalized by coating with adhesive mussel-inspired polydopamine (PD) facilitated the adsorption of RE-1 silencing transcription factor (REST)-directed siRNAs and subsequently resulted in knocked down REST for 5 days in neural progenitor cells (NPCs) [88]. Compared with the simple non-specific random adsorption approaches, the functionalization of fibrous interfaces to coordinate the interactions between gene vectors and fiber surfaces readily inhibited the aggregation of vectors deposited on the surfaces and thus allowed for homogeneous spreading of the vectors, thereby possibly promoting efficient transfer of the vectors across the cellular membrane [17].

#### Spatially patterned or localized gene delivery on fibrous surfaces

**Figure 5:**
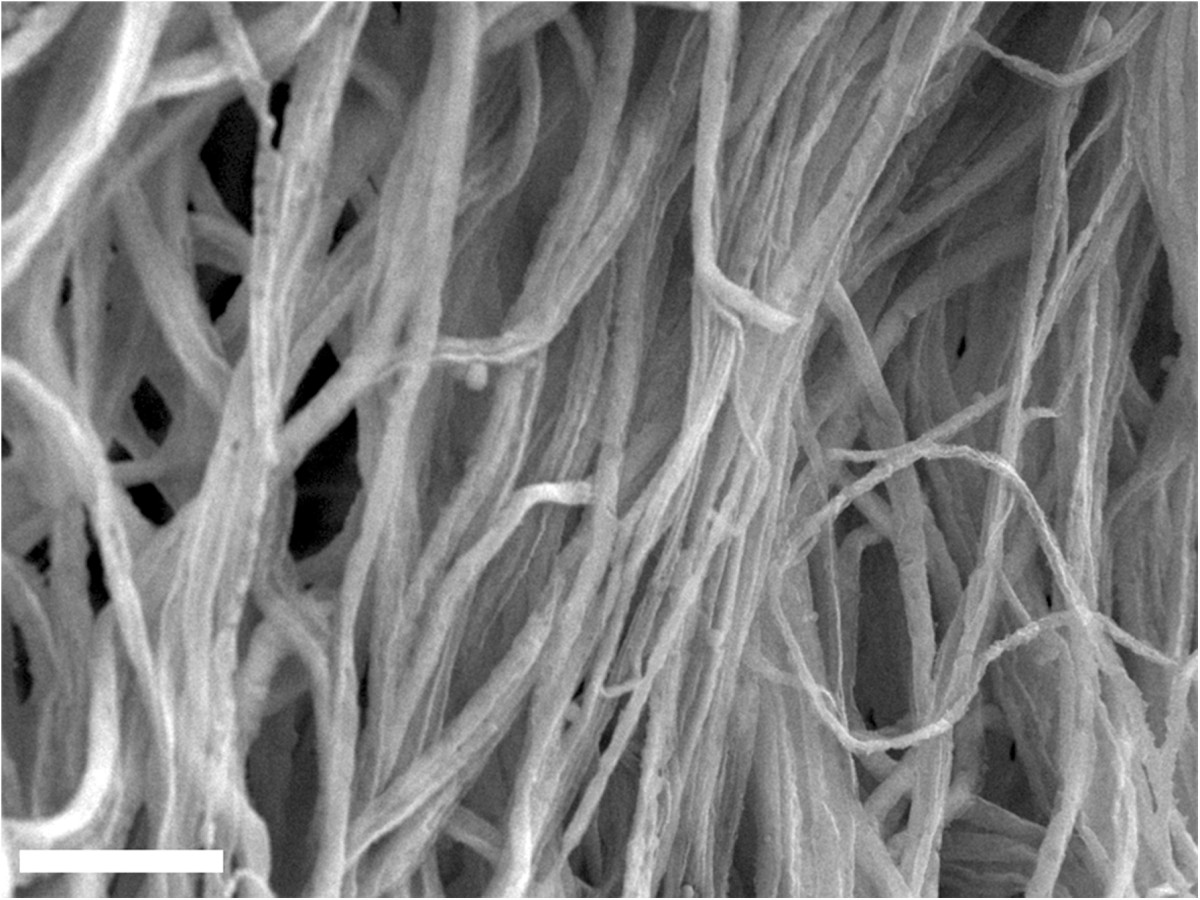
**SEM image of uniaxially patterned PCL nanofibers fabricated by utilizing rotating mandrels.** The scale bar indicates 10 μm. The image was adapted with permission from [33]. Copyright 2014 American Chemical Society.

One of the key unique characteristics of electrospinning compared with other fabrication tools is that it can readily produce uni-axially aligned or patterned fibrous structures (Figure 5). The uniaxial alignment of polymeric nanofibers has been widely used in numerous tissue engineering applications that require specialized tissue orientations or patterns, such as nerve or muscle regeneration [89–92]. Numerous fibrous devices demonstrating morphological orientations with specialized patterns have already been developed mainly through the use of approaches such as collector modification [35, 93] or electromagnetic methods [94], but a few patterned matrices have been employed for gene delivery templates. Recently, Lee et al. developed highly fluffy three-dimensional, uni-axially patterned PCL nanofibrous matrices using co-axial electrospinning on a rotating mandrel followed by a selective leaching process [33]. Human embryonic kidney cells were cultured on the patterned fibers where AAV vectors encoding for green fluorescent protein (GFP) were pre-immobilized by non-specific adsorption. Consequently, the patterned-physical guide arranged the GFP-expressing cells uni-axially, demonstrating the potential of this platform tool to induce spatially patterned gene expression.

The modification of either fibrous surfaces or gene vectors can allow spatially patterned or localized gene delivery on the fibrous matrices, and these matrices can possibly be used for patterned tissue regeneration [95–97]. Covalently coupling gelatin onto poly(D,L-lactide) (PDLLA) via sequentially involving an aminolysis reaction and Schiff base formation created fibrous matrices with gradients in HAp contents, which ultimately led to gradients in the plasmid DNA content through the mineralization process [98]. The gradient deposition of plasmid DNA on the modified surfaces induced spatially patterned gene transfection, which corresponded to the graded patterns of cell distribution as well as osteoblastic differentiation [98]. Additionally, the chemical alterations of the gene vector itself played a key role in inducing spatially patterned gene expression on fibrous PCL matrices [60]. The complexation of negatively charged AAV vectors with positively charged catechol-conjugated PEI generated adhesive viral vectors, facilitating the immobilization of AAV vectors onto the fibrous PCL matrices due to their stickiness [60]. Adjusting the stickiness properties of AAV vectors regulated the gene delivery efficiencies and optimized the increase in gene transfer compared with that for non-modified AAV vectors. The resultant sticky viral system aided in the spatially patterned deposition of viral vectors via a simple pipette drawing technique and soft lithography. In principle, creating the concentration gradients of gene vectors in a localized region can induce patterned gene expression or oriented growth factor generation that corresponds to the gradients of the gene vectors; thus, this strategy can offer basic tools to promote patterned tissue regeneration. Similarly, a recent study demonstrated that manipulating the adhesive properties of surfaces can work as a key design parameter to control the release profiles of immobilized gene vectors for inducing sustained gene expression; this strategy can be further translated into electrospun nanofibers [99].

## Potential applications of electrospun nanofibers that release gene vectors

### Tissue engineering

Owing to both their versatile capabilities that can be used to coordinate the release profiles of gene vectors and their ECM-analogue nature (Figure 1), gene vector-eluting electrospun nanofibrous structures have primarily been used for tissue regeneration approaches. In this combinatorial gene delivery approach using electrospun fibers as tissue engineering scaffolds, the spatially or temporally regulated secretion of tissue inductive growth factors from cells can initiate either autocrine or paracrine effects to stimulate cellular processes for tissue formation; the cells are transfected or transduced by gene vectors carrying the specific genes [7, 8, 18, 95]. Compared with the direct protein delivery approach, this approach allows for the sustained secretion of fresh growth factor proteins from the cells, and this secretion can play a key role in maintaining the stability of the proteins, potentially maximizing the efficacy of tissue formation [7, 8, 100]. Additionally, the capabilities of inducing localized gene expression adjacent to polymeric systems can further increase the efficacy to promote tissue formation in a defined area [18]. Thus, electrospun nanofibers that release gene vectors have been employed as a platform scaffold for mediating the regeneration of tissue, such as bone [56, 77, 82], skin [66, 76, 84–86], blood vessels [65, 78, 83, 87], and nervous system tissues [88].

#### Bone tissue engineering

**Figure 6:**
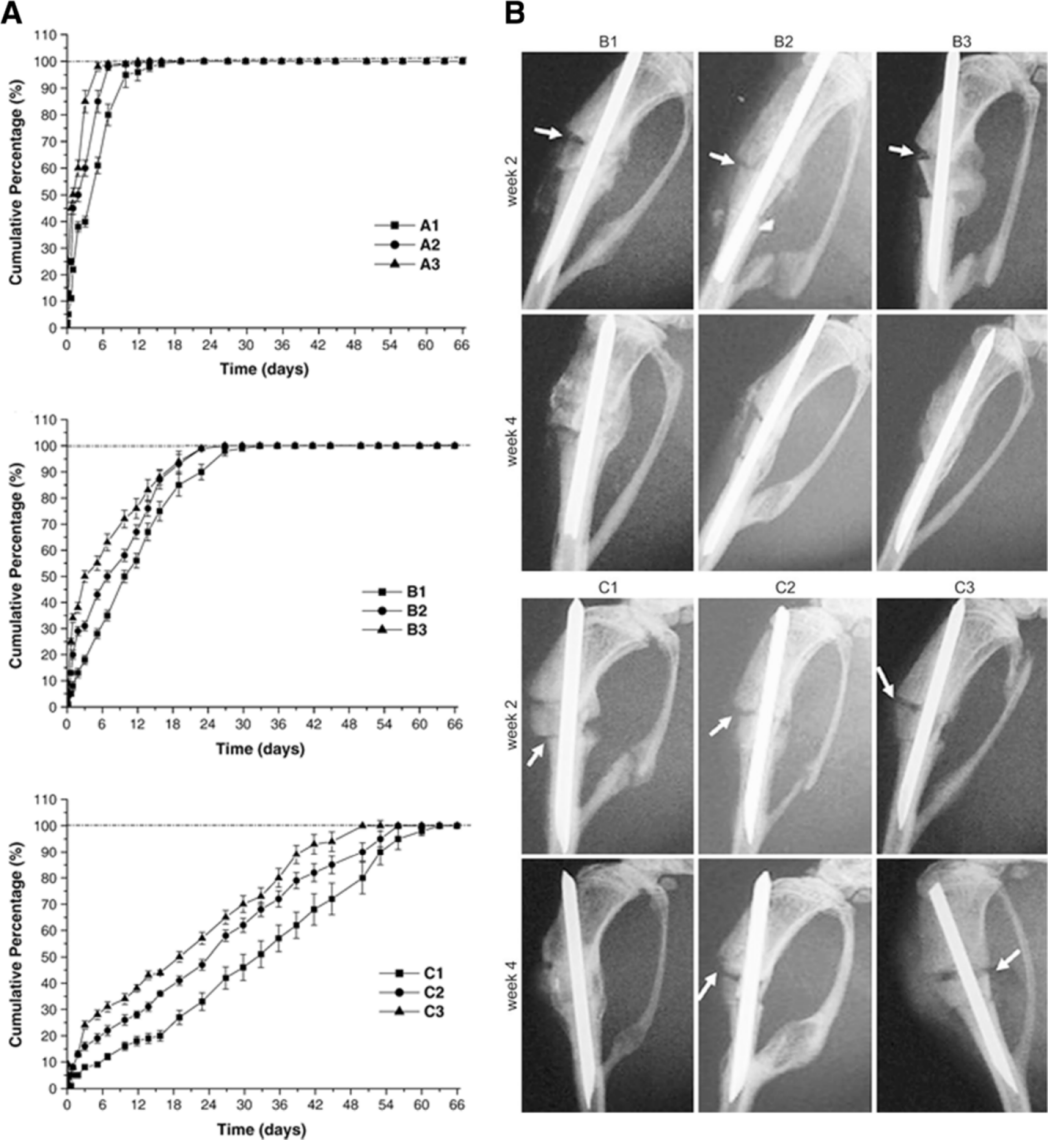
**BMP-2 plasmid loaded electrospun scaffolds for bone tissue engineering. (A)** *In vitro* release curve of three groups of scaffolds [56], Copyright 2007. Reproduced with permission from Elsevier. **(B)** Radiographs of nude mice tibias after 2 and 4 weeks of implantation of scaffolds. Bone fragment without implantation of any scaffold is denoted as control and white arrows identify bone defects [82], Copyright 2009. Reproduced with permission from Elsevier. (Group A: PLGA/HAp composite fiber with naked DNA coated outside, Group B: PLGA/HAp composite fiber with DNA-loaded chitosan nanoparticles coated outside, Group C: PLGA/HAp composite fiber with DNA-loaded chitosan nanoparticles encapsulated inside. The number indicates HAp contents in composite. X1: 0/100, X2: 5/95, X3: 10/90 (HAp/PLGA w/w%)).

In addition to the ability of electrospun fibers to precisely mimic bone ECM, these fibers have large surface-to-volume ratios, allowing vascularization across the newly produced tissues within fibrous structures; these features provide strong rationales for the use of nanofibers as a guide to regenerate bone tissues [101]. Bone morphogenetic protein 2 (BMP-2) is a representative osteoinductive protein that plays an important role in directing the cellular processes that regenerate bone or cartilage [82]. Wang et al. fabricated electrospun scaffolds comprised of a PLGA/HAp composite; these scaffolds released plasmid DNA encoding for BMP-2 to promote bone tissue formation *in vitro*[56] and *in vivo*[82]. The sustained release of chitosan/DNA-BMP-2 complexes (Figure 6A) localized the BMP-2 expression at the region adjacent to the PLGA-HAp fibrous matrices. Consequently, the coordination of the release modes of the chitosan/DNA-BMP-2 complexes regulated both the transfection efficiencies and the cellular viabilities [56], ultimately resulting in improved healing of segmental bone defects in mouse tibias (Figure 6B) [82]. Additionally, the delivery of plasmid DNA encoding a transcription factor, which regulates the cascades for the expression of multiple endogenous genes or for intracellular signals, can act as a key tool to promote bone tissue formation. The PCL nanofiber-mediated delivery of liposomes programmed to up-regulate RUNX2, a factor that induces cellular differentiation into the osteoblast phenotype, increased the osteogenic differentiation of hBMSCs [77]. As previously mentioned, the electrospun fibers aided in the reduction of the aggregation of liposome-RUNX2 and the cellular toxicity, leading to improved delivery efficiencies and cellular differentiation. Eventually, orchestrating the osteogenesis, angiogenesis, and inflammation at injured sites will be a crucial factor to repair or form new bone tissues functionally, which is currently a critical challenge [102]. Thus, creating synergistic effects from multiple factors, including osteogenic factors (e.g., transforming growth factor-β (TGF-β or growth differentiation factor (GDF)), angiogenic factors (e.g., VEGF or platelet-derived growth factor (PDGF)), and inflammatory inhibitory factors, through coordination of the delivery modes of these factors from electrospun fibers would be the next challenge in bone tissue engineering. Additionally, many advanced electrospinning technologies capable of readily manipulating pore sizes, mechanical properties, and three-dimensional morphologies would be required to further improve the efficiency of bone tissue engineering [103].

#### Skin tissue engineering

**Figure 7:**
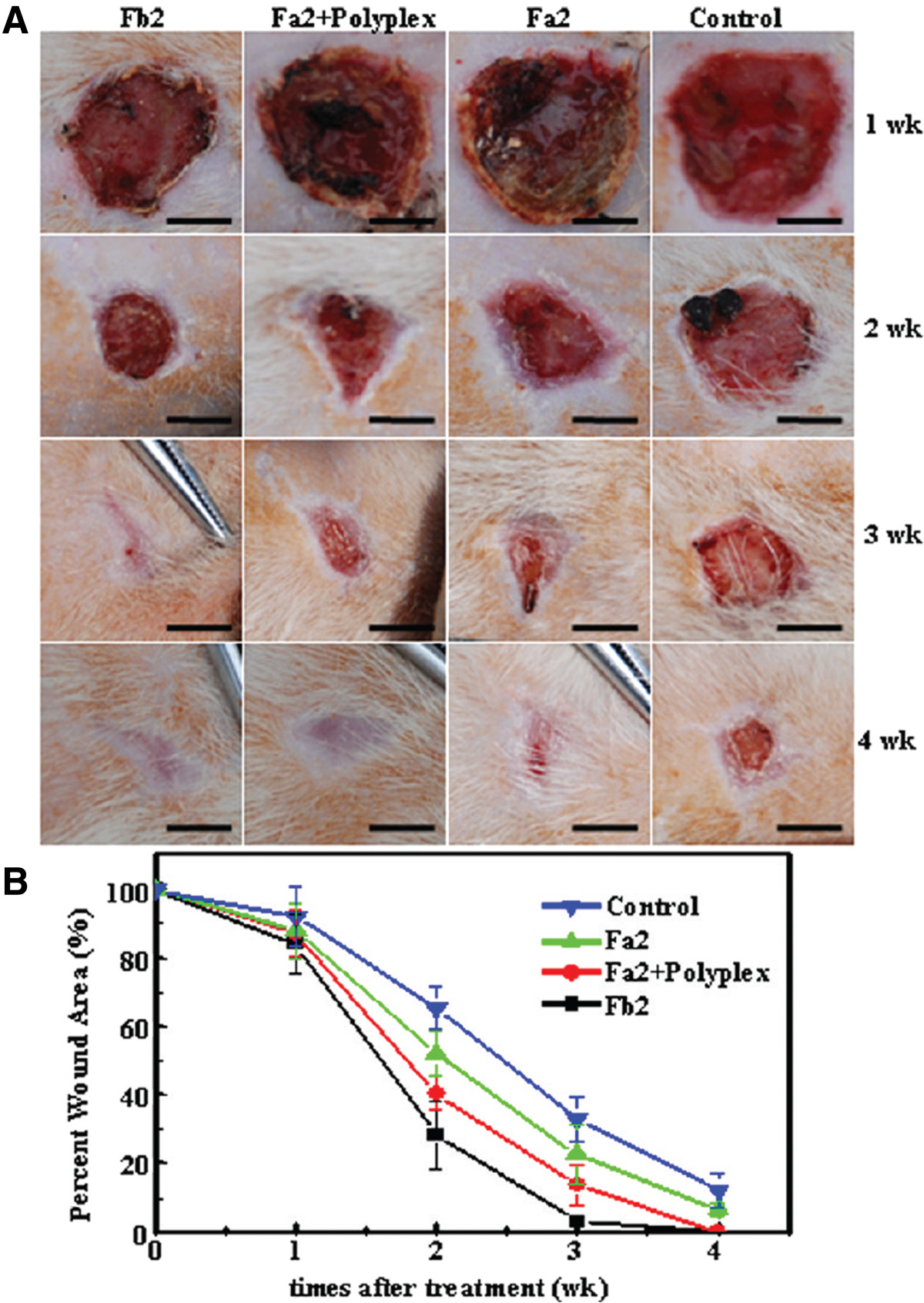
**Improved skin regeneration by electrospun fibers with plasmid bFGF polyplex. (A)** The representative images of skin wounds after treatment with Fb2 (pbFGF polyplex, blend), Fa2 (without pbFGF) and Fa2 + Polyplex (pbFGF polyplex infiltrated Fa2) fibrous mats for 1, 2, 3, and 4 weeks, using untreated wound as control. Bars represent 10 mm. **(B)** Wound areas at different time points after treatment (n = 10). Reprinted with permission from [84]. Copyright 2012 American Chemical Society.

Skin tissue engineering has emerged as a powerful means to promote wound healing, a sophisticated cellular process that can be achieved by coordinating hemostasis, inflammation, epithelialization, angiogenesis, and collagen synthesis [104–106]. Importantly, highly porous electrospun nanofibrous sheets, which provide suitable environments for precisely mimicking the morphology of skin ECM components [105], have been considered as robust templates for promoting skin tissue regeneration. Kim et al. developed matrix metalloproteinase (MMP)-responsive electrospun nanofibrous matrices that release plasmid DNA encoding that encodes human epidermal growth factor (hEGF) to generate skin tissue in diabetic ulcer animal models [66, 85, 86]. The MMP-cleavable linker was conjugated to the amine group on the linear PEI, which was present on the surface of the fibrous matrices to anchor the plasmid DNA-hEGF through electrostatic interactions. Subsequently, the exposure to the MMPs, which are inherently overexpressed in diabetic ulcers, cleaved the DNA-hEGF for subsequent localization of the gene expression in a controlled manner [66]. The resultant electrospun fiber systems were used to increase the expression levels of hEGF in primary human dermal fibroblasts (HDFs) [85], ultimately accelerating the wound healing rates in animal models [66, 85]. In a different study, the suppression of MMP in diabetic animal models through treatment with siRNA-decorated nanofibrous sheets for 7 days dramatically increased neo-collagen accumulation at dorsal wound sites, which subsequently triggered improvements in the wound recovery rates [86]. Additionally, the sustained delivery of PEI/DNA complexes encoding basic fibroblast growth factor (bFGF) from core-sheath fibers that were generated using PELA/PEG blends significantly increased the efficiency of transfecting mouse embryonic fibroblasts, resulting in improved skin regeneration in dorsal wound diabetic rat models (Figure 7) [84]. The accumulation of multiple layers of keratinocyte growth factor (KGF)-encoding plasmid DNA on the electrospun PLA/PCL fibers achieved robust re-epithelialization, keratinocyte proliferation, and granulation responses, thereby inducing full-thickness wound recovery in mouse cutaneous wound models [76]. Taken together, these findings show that the spatiotemporally regulated delivery of multiple inducible factors [106] and the use of *ex vivo* stem cell transplantation [107] with electrospun fibrous structures would be a successful strategy for constructing dermal or epidermal tissue layers.

#### Vascular tissue engineering

Both endothelial cell lining and smooth muscle cell layers on the luminal surface of vessel architectures have been regarded as crucial design factors to explore in vascular tissue engineering [51, 108]. Additionally, providing both the secretion of an angiogenic growth factor and a physical guide are of great importance in vascular regeneration. Thus, gene vector-releasing electrospun fibers with an ECM-analogue, which acts as a support for the endothelial cells to proliferate, can provide efficient physical cues for vascular tissue engineering [109]. Subcutaneous implantation of PELA fibrous scaffolds eluting dual plasmids encoding VEGF and bFGF accelerated the maturation of blood vessels compared with single plasmid DNA delivery approaches [65, 83]. Non-specific adsorption of PEI/DNA complexes encoding for VEGF for the subsequent substrate-mediated delivery of the vectors resulted in the robust expression of VEGF in H9C2 myoblast cells and triggered local angiogenesis adjacent to the electrospun fibers, demonstrating the future potential of this method for the treatment of myocardial infarction [87]. Importantly, an abnormal tissue response in conjunction with excessive vascularization may cause the failure of tissue grafts. Thus, suppressing the up-regulation of angiogenic factors to reduce the intimal hyperplasia may sometimes be required to produce functional tissue grafts with suitable vascularized structures. The amine-functionalized poly(ethylene terephthalate) (PET) electrospun fiber-mediated delivery of PEI/siRNA-thrombospondin-2 (TSP-2), an anti-angiogenic matricellular protein, was designed to lead to TSP-2 knockdown in primary human aortic smooth muscle cells (AoSMCs), eventually improving the biocompatibility between implanted materials and host tissues [78]. Importantly, the sequential delivery of multiple gene vectors in a spatiotemporal manner may be appropriate to regenerate blood vessels composed of double layers, including vascular endothelial cells in the interior layer and vascular smooth muscle cells in the outer layer [51]. Thus, mimicking the double layered structures with highly porous fibrous structures that can release multiple gene vectors capable of stimulating cells individually in each layer will be useful to maximize the efficacy of vascular grafts, as well as for vascular tissue engineering.

#### Neural tissue engineering

**Figure 8:**
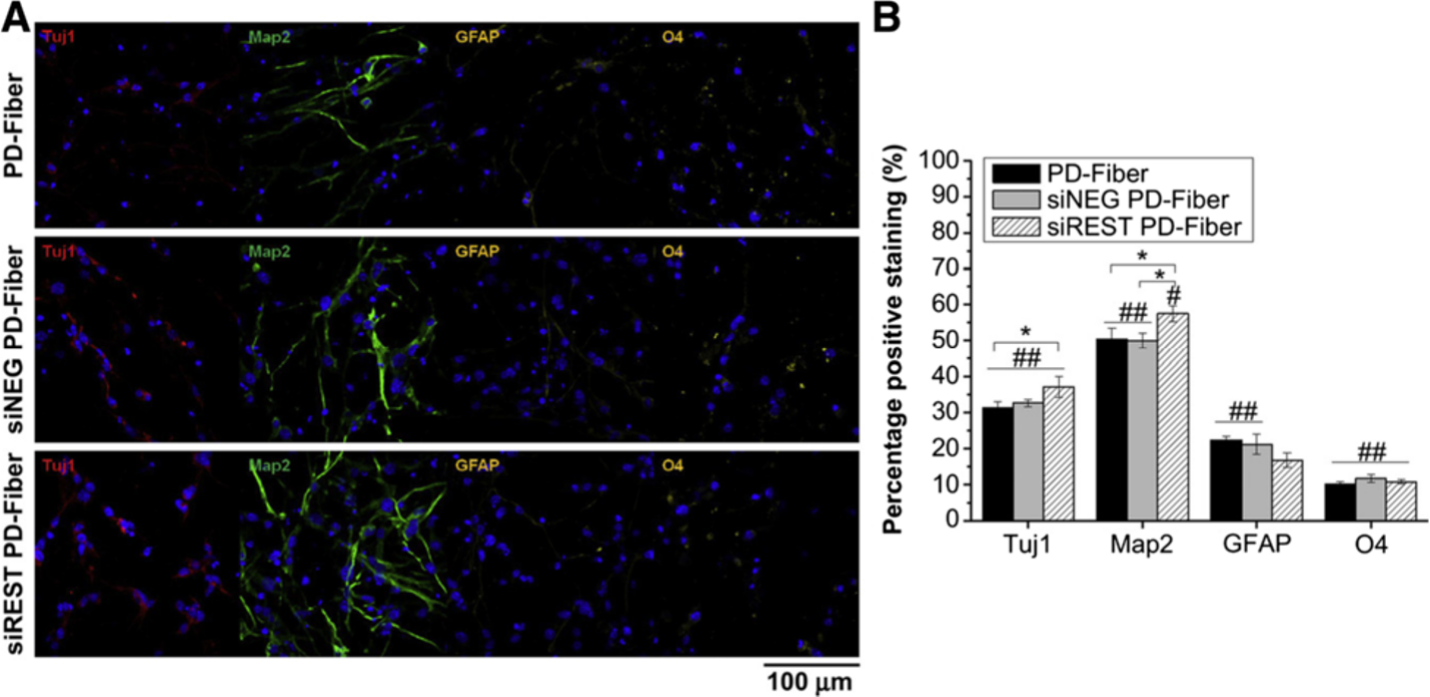
**Immunofluorescence analysis of NPCs differentiated on plain, siNEG PD-fiber and siREST PD-fiber for 7 days. (A)** Immunostaining for Tuj1 (early neuronal marker, red), Map2 (late neuronal marker, green), GFAP (glial marker, yellow) and O4 (glial marker, yellow). Nuclei were counterstained with DAPI (blue). **(B)** Quantification of immunostaining results showing percentage positive staining of various neural and glial cell markers for NPCs cultured on all PD-fiber samples. *indicates p < 0.05 (ANOVA). # and ## indicate p < 0.05 and p < 0.01 (ANOVA) when PD-fiber samples were compared to respective PD-film samples. Mean ± SE, n = 3 [88], Copyright 2013. Reproduced with permission from Elsevier.

Employing neural stem cells, which have the capabilities of self-renewal as well as neuronal differentiation, and providing patterned physical guidance along with biochemical cues (e.g., growth factors or genes) have been regarded as pivotal factors to induce neural regeneration [110, 111]. Thus, the feasibility of adjusting the orientation or topographical changes of electrospun fibers during the electrospinning process facilitated the use of these fibers as versatile scaffolds to guide neuronal cell growth or direct neuronal differentiation on their surfaces [112, 113]. The topological effect of nanofibers along with the knockdown of REST in NPCs synergistically promoted the neuronal differentiation of NPCs on the adhesive mussel-inspired PD-coated PCL fibrous surfaces while also reducing astrocytic and oligodendrocytic differentiation (Figure 8) [88]. The physical alignment of the electrospun fibers that release neuronal inducible factors at nerve lesion sites can guide the neurite outgrowth along with the orientation; these capabilities will be required for the functional recovery of injured neurons [95, 96]. Therefore, the ability to easily produce patterned structures using electrospun fibers will lead to extensive applications of these fibers for numerous incurable chronic neurodegenerative diseases.

### Other applications

Electrospun fibers have been recently employed in a stem cell study or a cancer therapy approach in addition to their use in tissue engineering applications. Fei et al. fabricated a nanofiber-based sandwich electroporation (NSE) device capable of *in situ* gene transfection in mouse embryonic stem cells [114]. Unlike a conventional bulk electroporation, this NSE system did not require the removal of adherent cells from the substrate, thereby improving cellular viability and ultimately enhancing transfection efficiency. This study demonstrated that gene vector-releasing electrospun nanofibers can be potentially employed as powerful templates to elucidate stem cell behavior or biology. Meanwhile, Achille et al. utilized electrospun PCL fibers as a gene carrier for the treatment of breast cancer [115]. A plasmid encoding short hairpin RNA (shRNA) suppressing the level of the cell cycle specific protein, cyclin-dependent kinase 2 (Cdk2), was blended with PCL solutions to construct highly porous electrospun fibers. The interruption of the cell cycle by the silencing effect of the shRNA, which was gradually released from the electrospun PCL fibers, robustly led to the suppression of the proliferation of breast cancer cell lines. This study also indicates that the combination of gene delivery with electrospun nanofibers, which can create ECM-analogous environments and tunable gene delivery in a spatial and temporal manner, can provide an alternative powerful means to specifically target a variety of cancer cells.

## Conclusions and challenges

Gene therapy has demonstrated increasing promise for treating a variety of human diseases, including inherited or acquired disorders, infectious diseases, tissue loss, and organ failure. The development of highly efficient gene delivery systems that can deliver a gene of interest safely to particular target cells has been always regarded as a large hurdle that must be cleared for the further advancement of gene delivery technologies. Electrospun fibers have highly advantageous characteristics, including ease of production, an ECM-analogue nature, a broad range of choices for materials, the feasibility of producing structures with varied physical and chemical properties, and large surface-to-volume ratios. Because of these characteristics, electrospun nanofibers have recently been highlighted as versatile and powerful templates that can be applied to numerous biomedical fields. Therefore, the integration of gene delivery with electrospun nanofibers is a highly promising strategy to improve gene delivery for a broad range of applications.

For further innovation of approaches that combine gene delivery and electrospun nanofibers, there are several critical challenges, especially improving the structural aspects of electrospun nanofibers. Electrospinning typically produces flat two-dimensional sheet-like meshes, which may not fully represent the three-dimensional extracellular environments in the body. These structural characteristics may limit the ease of application of electrospun nanofibers to many biomedical fields. Numerous studies have created three-dimensional electrospun nanofibers, typically by modifying collectors or by utilizing an extra apparatus [116]. Additionally, engaging the structural flexibility of electrospun nanofibers, which is required to precisely mimic the various shapes of tissues or organs, may be another challenge that must be addressed. Recently, clay-like moldable electrospun nanofibers were created by adjusting the electric repulsion between different materials used to produce the core-sheath layers and then selectively removing sacrificial fibers in the sheath layer [33]. Owing to their moldable clay-like properties, any desired forms, such as the human nose, a ball, or tubes, could be manually shaped. The combination of the clay-like electrospun fibers with viral gene delivery resulted in a high level of gene expression throughout the entire fibrous structure [33]. Adapting micro- and nanofabrication technologies to generate electrospun nanofibrous structures to mimic the spatial and temporal control of the expression of multiple genes in tissues or organs is another challenge that must be addressed. Electrospun nanofibers are powerful candidates for mediators of the spatiotemporal delivery modes of multiple gene vectors because their structure makes it possible to generate patterned fibers and core-sheath structures. However, the mechanical strength of electrospun fibers, which is inherently lower than that of existing polymeric scaffolds, must be reinforced to resist cellular contractile forces upon implantation and to maintain the structural integrity of the scaffolds. Finally, the development of non-invasive electrospun nanofibers, such as injectable formulations, would contribute to the increasing promise of fibrous systems as spatial templates. Overall, further innovation in improving the structures of electrospun nanofibers will be pivotal to extend their use to a variety of biomedical applications, especially gene delivery applications.

## Authors’ original submitted files for images

Below are the links to the authors’ original submitted files for images. Authors’ original file for figure 1 Authors’ original file for figure 2 Authors’ original file for figure 3 Authors’ original file for figure 4 Authors’ original file for figure 5 Authors’ original file for figure 6 Authors’ original file for figure 7 Authors’ original file for figure 8

## Abbreviations

AAV: Adeno-associated virus
Ad: Adenovirus
AoSMC: Aortic smooth muscle cell
bFGF: Basic fibroblast growth factor
bFGF: Basic fibroblast growth factor
BMP-2: Bone morphogenetic protein-2
Cdk2: Cyclin-dependent kinase 2
CMV: Cytomegalovirus
ECM: Extracellular matrix
EEP: Ethyl ethylene phosphate
ELP: Elastin-like polypeptides
GDF: Growth differentiation factor
GFP: Green fluorescent protein
HAp: Hydroxyapatite
HA-PEI: PEI conjugated with hyaluronic acids
hBMSC: Human bone-marrow-derived mesenchymal stem cell
HDF: Human dermal fibroblasts
hEGF: Human epidermal growth factor
HFP: Hexafluoro-2-propanol
KGF: Keratinocyte growth factor
LBL: Layer-by-layer
MMP: Matrix metalloproteinase
NPC: Neural progenitor cell
NSE: Nanofibers-based sandwich electroporation
PBAE: Polycationic poly(β-amino ester)
PCL: Poly(caprolactone)
PCLEEP: Poly(caprolactone-co-ethylethylene phosphate)
PD: Polydopamine
PDGF: Platelet-derived growth factor
PDLLA: Poly(D,L-lactide)
PEG: Poly(ethylene glycol)
PEI: Poly(ethylenimine)
PELA: Poly(D,L-lactide)-poly(ethylene glycol)
PET: Poly(ethylene terephthalate)
PLA: Poly(lactic) acid
PLCL: Poly(L-lactide-co-ϵ-caprolactone)
PLGA: Poly(D,L-lactide-co-glycolide)
REST: RE-1 silencing transcription factor
siNEG: Negative siRNA
RUNX2: Runt-related transcription factor 2
shRNA: Short hairpin RNA
siRNA: Small interfering RNA
TGF-β: Transforming growth factor-β
TSP-2: Thrombospondin-2
VEGF: Vascular endothelial growth factor.
