# Supplementary figures and images for: Electrospun nanofibers as versatile interfaces for efficient gene delivery
Source: J Biol Eng. 2014 Dec 9;8:30. doi: 10.1186/1754-1611-8-30 (PMC4414388; doi:10.1186/1754-1611-8-30)

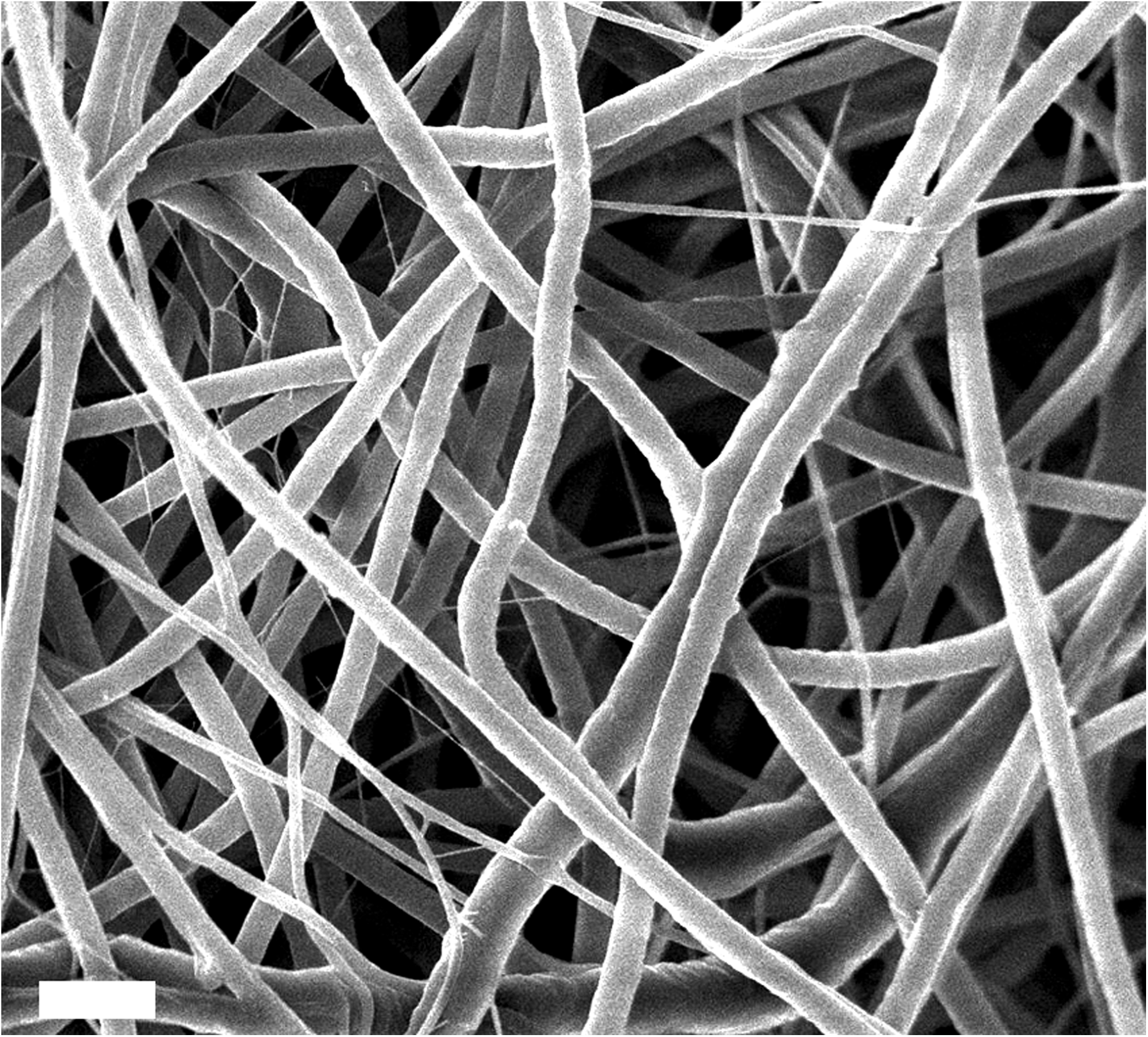

Supplement: Supplementary file 1 — Authors’ original file for figure 1 [file 13036_2014_164_MOESM1_ESM.tif]

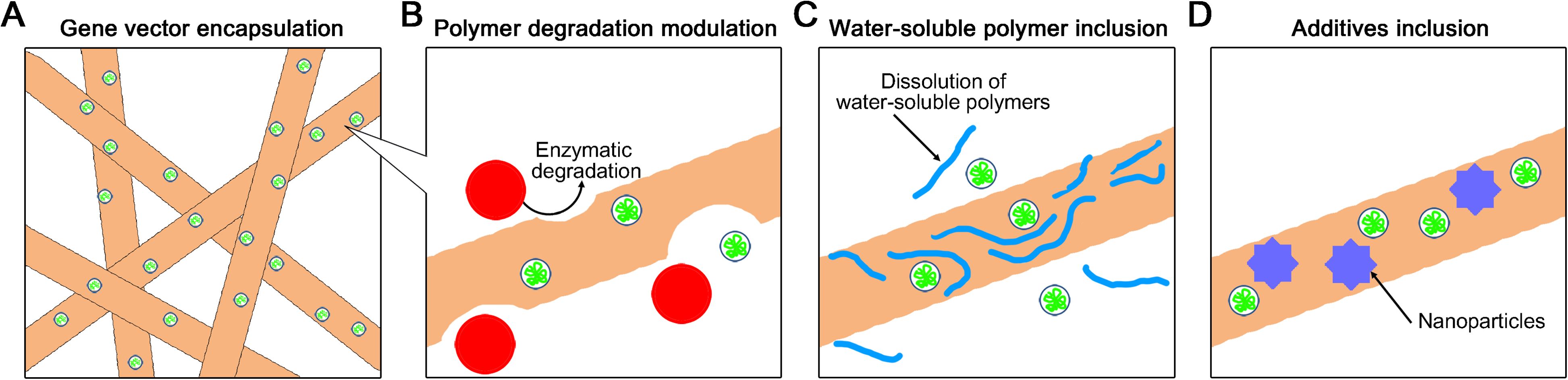

Supplement: Supplementary file 2 — Authors’ original file for figure 2 [file 13036_2014_164_MOESM2_ESM.tiff]

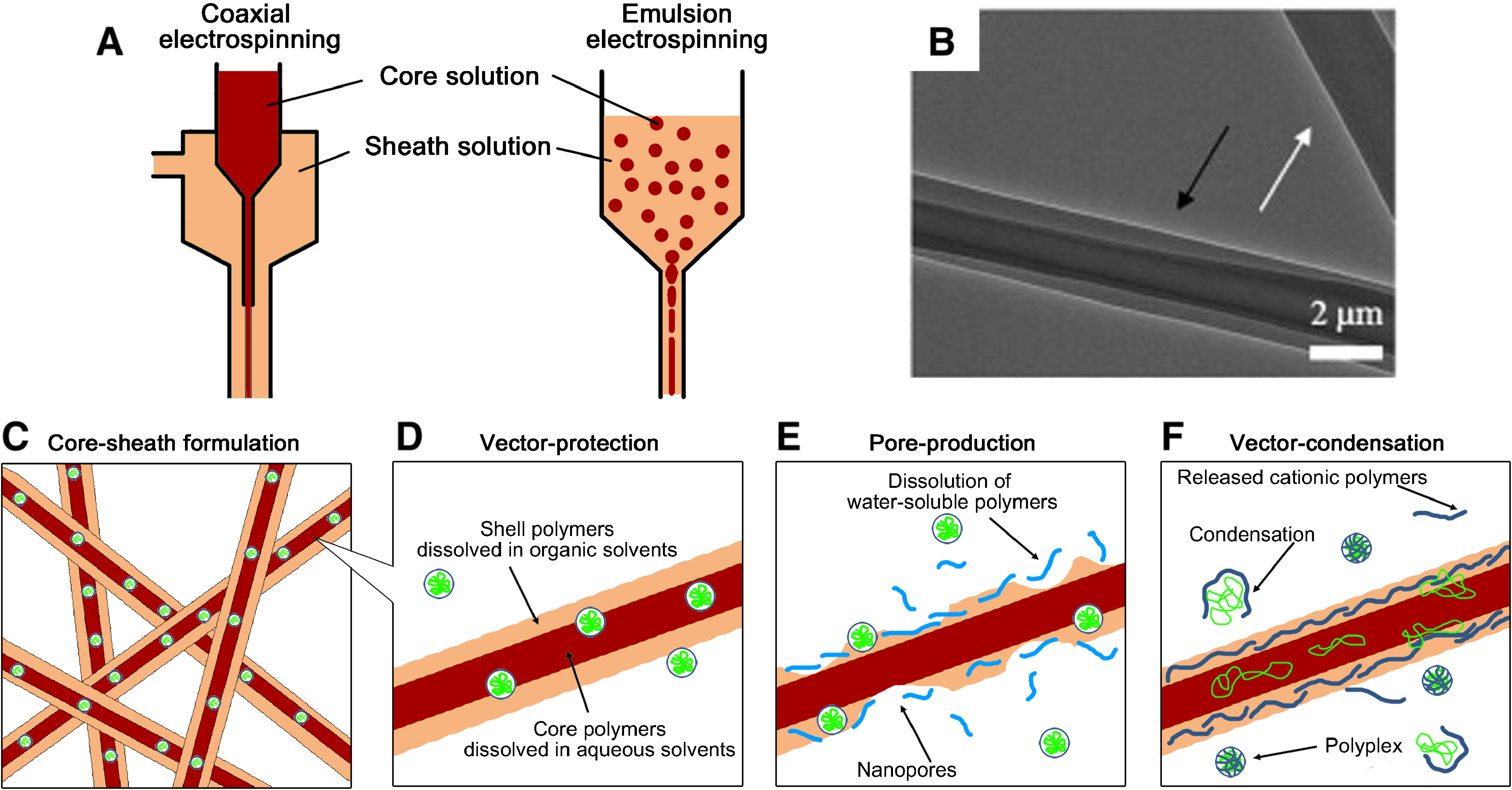

Supplement: Supplementary file 3 — Authors’ original file for figure 3 [file 13036_2014_164_MOESM3_ESM.tiff]

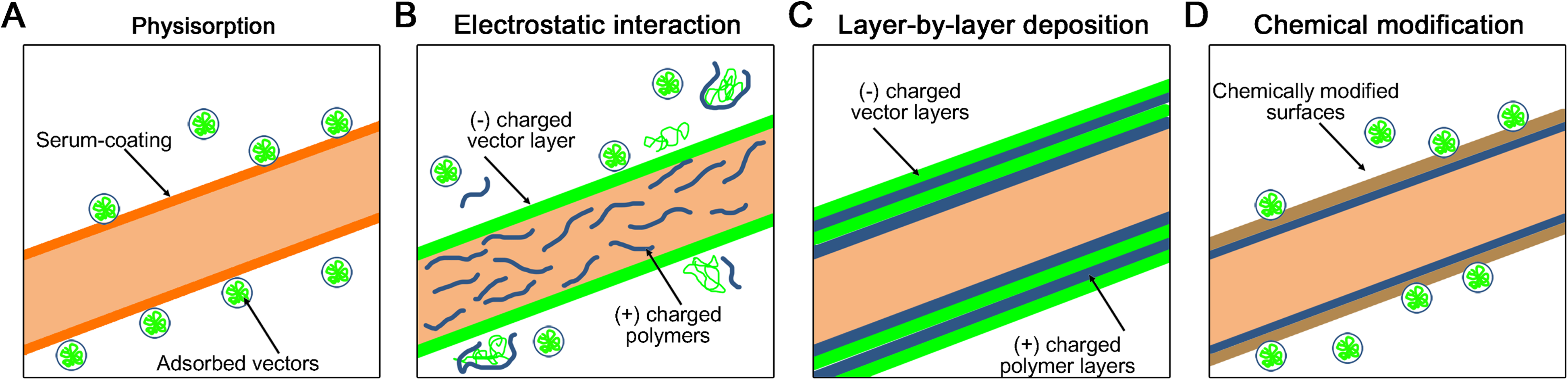

Supplement: Supplementary file 4 — Authors’ original file for figure 4 [file 13036_2014_164_MOESM4_ESM.tiff]

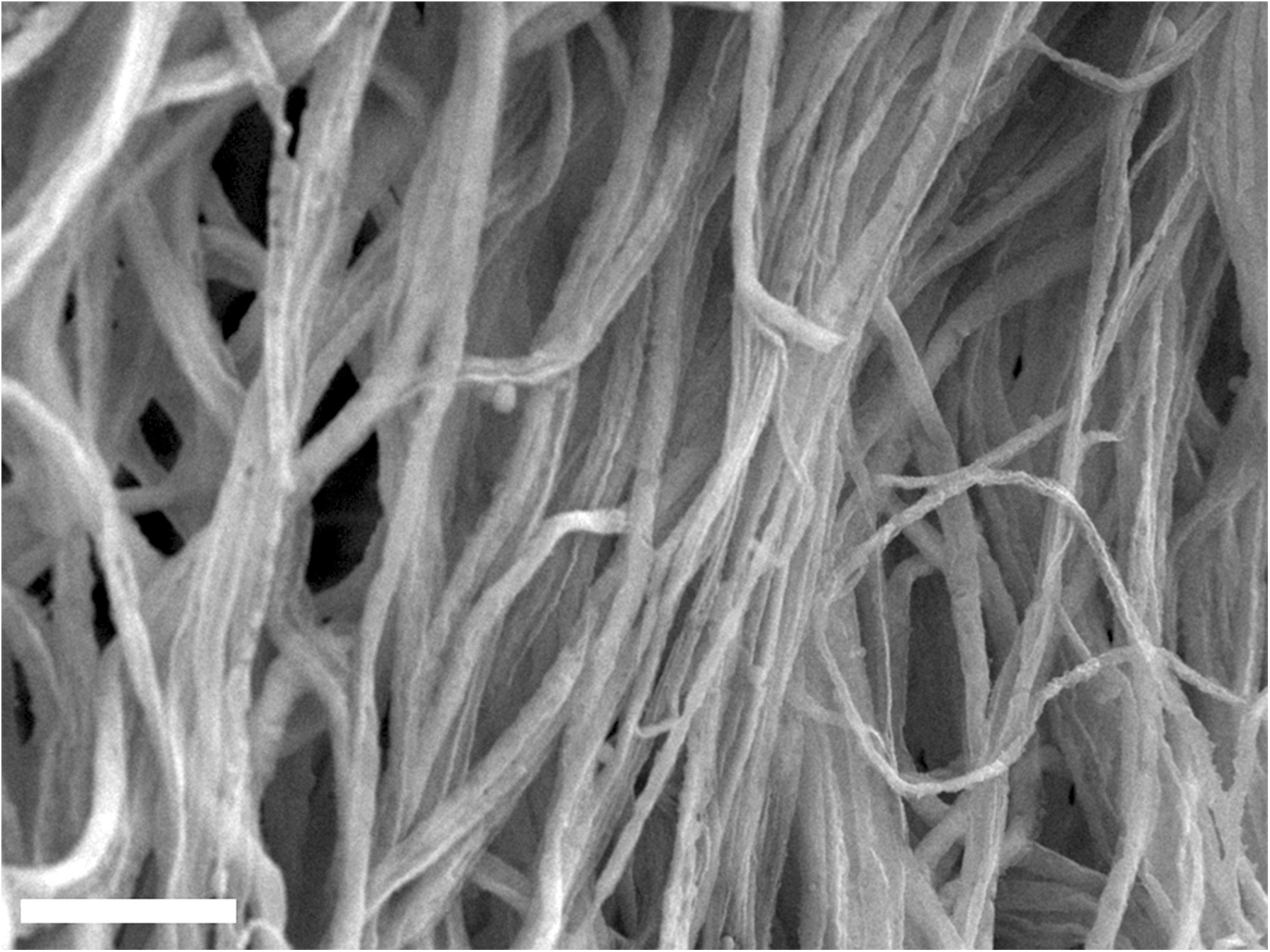

Supplement: Supplementary file 5 — Authors’ original file for figure 5 [file 13036_2014_164_MOESM5_ESM.tif]

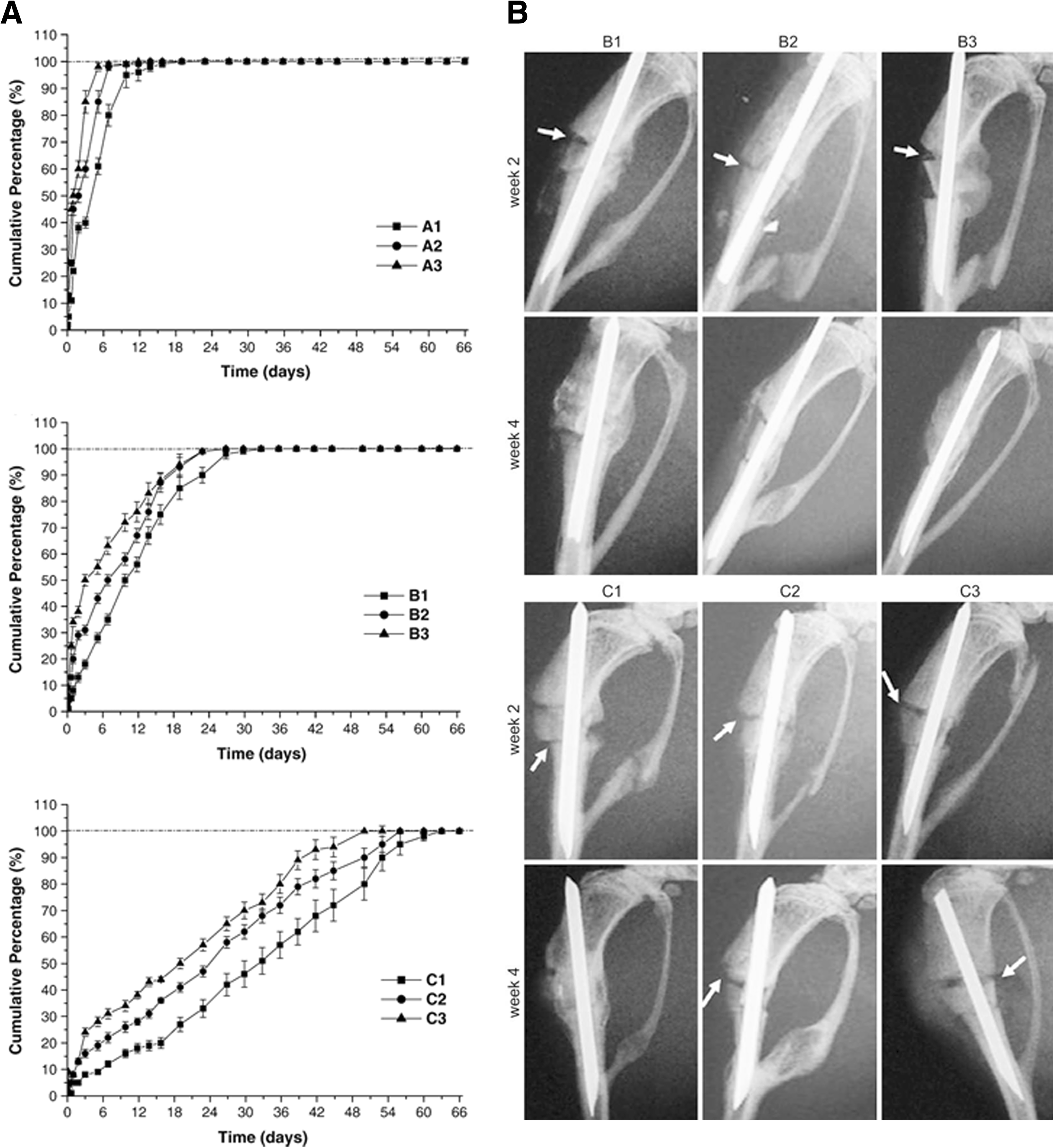

Supplement: Supplementary file 6 — Authors’ original file for figure 6 [file 13036_2014_164_MOESM6_ESM.tif]

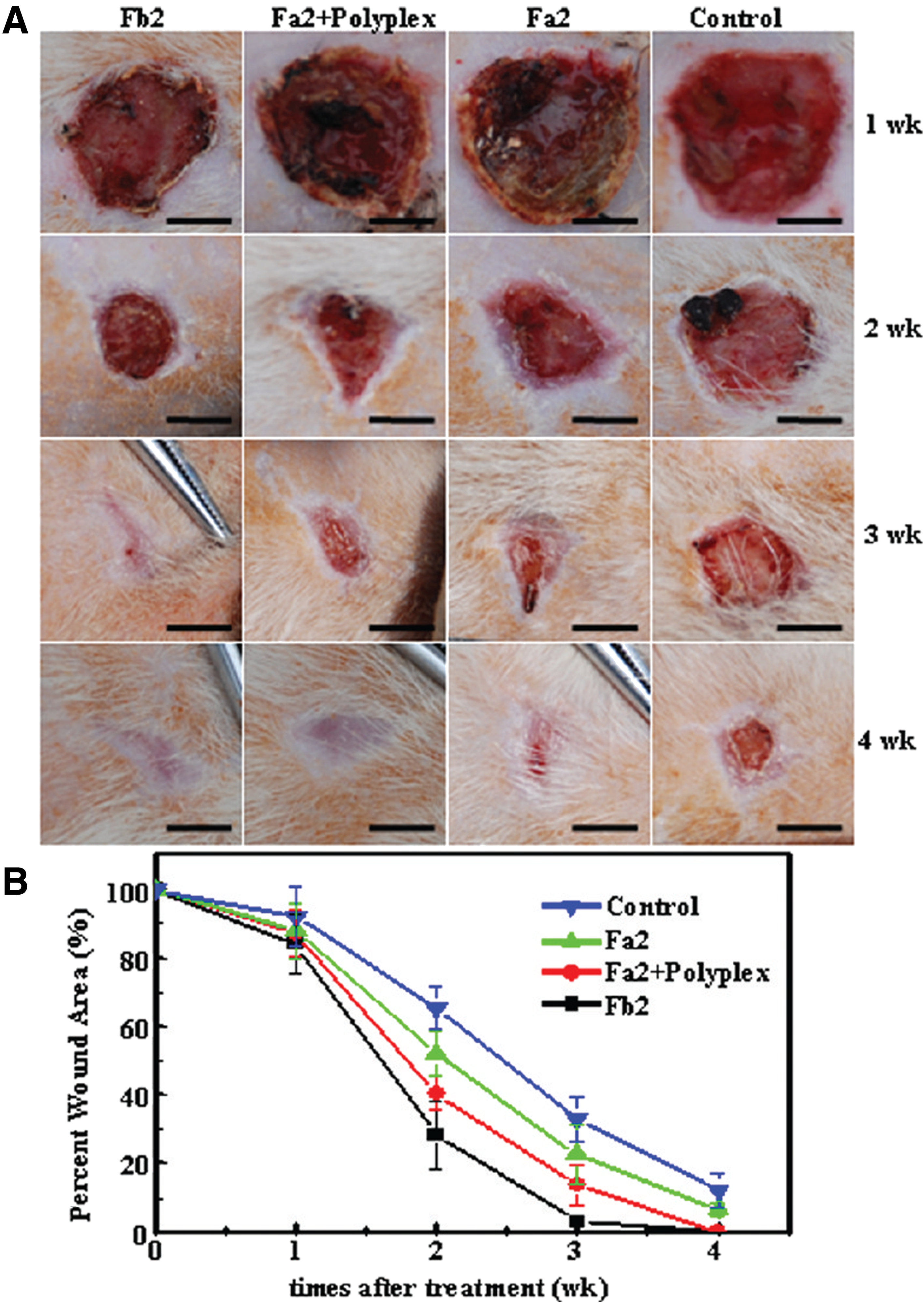

Supplement: Supplementary file 7 — Authors’ original file for figure 7 [file 13036_2014_164_MOESM7_ESM.tif]

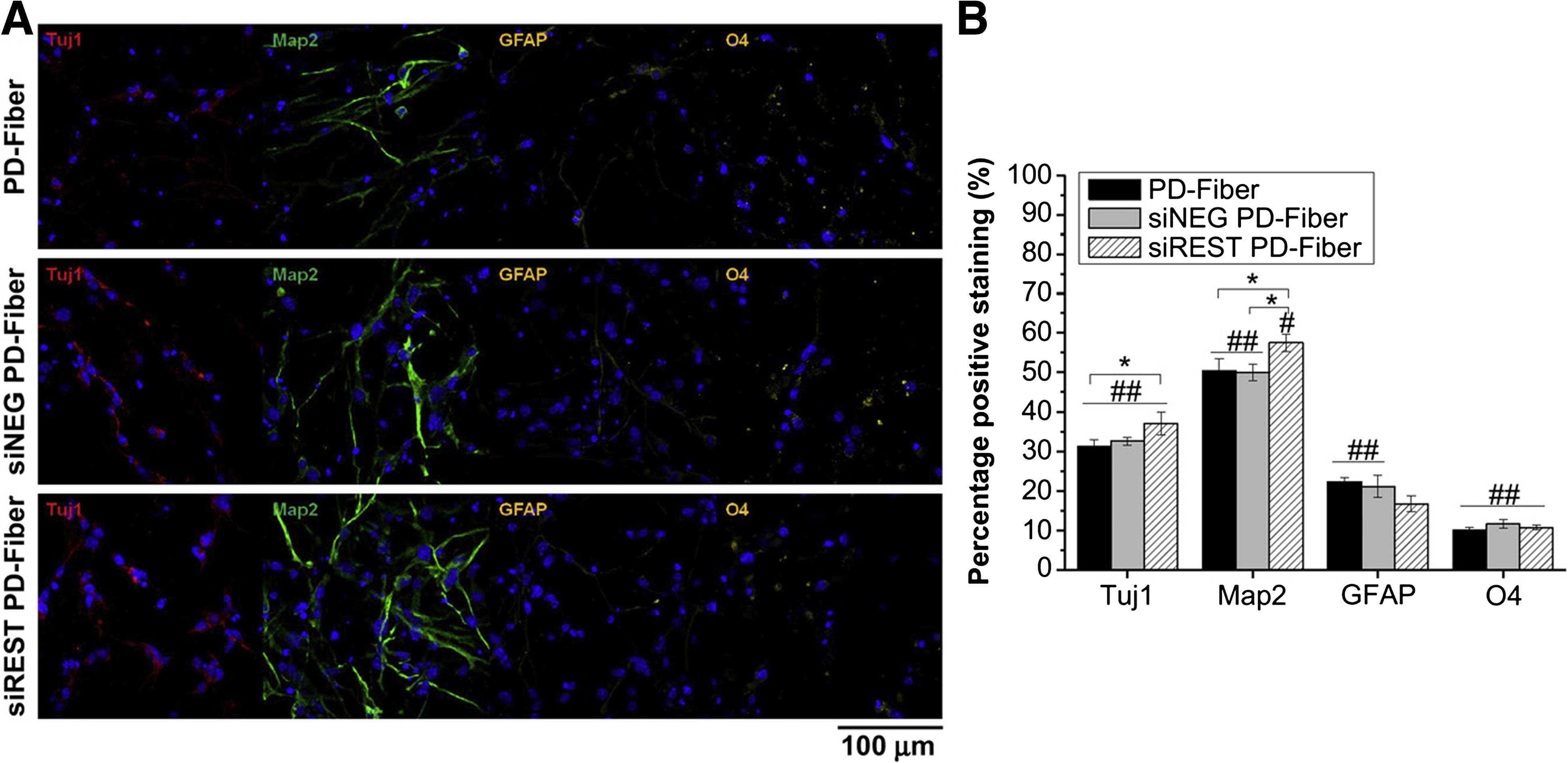

Supplement: Supplementary file 8 — Authors’ original file for figure 8 [file 13036_2014_164_MOESM8_ESM.tif]
